# Molecular Cloning and Functional Characterization of the Dual Oxidase (*BmDuox*) Gene from the Silkworm *Bombyx mori*

**DOI:** 10.1371/journal.pone.0070118

**Published:** 2013-08-02

**Authors:** Xiaolong Hu, Rui Yang, Xing Zhang, Lin Chen, Xingwei Xiang, Chengliang Gong, Xiaofeng Wu

**Affiliations:** 1 College of Animal Sciences, Zhejiang University, Hangzhou, China; 2 Institute of Cell Biology, Zhejiang University, Hangzhou, China; 3 National Engineering Laboratory for Modern Silk, Soochow University, Suzhou, China; Universidade Federal do Rio de Janeiro, Brazil

## Abstract

Reactive oxygen species (ROS) from nicotinamide adenine dinucleotide phosphate (NADPH) oxidases and their related dual oxidases are known to have significant roles in innate immunity and cell proliferation. In this study, the 5,545 bp cDNA of the silkworm *Bombyx mori* dual oxidase (*BmDuox*) gene containing a full-length open reading frame was cloned. It was shown to include an N-terminal signal peptide consisting of 28 amino acid residues, a 240 bp 5′-terminal untranslated region (5′-UTR), an 802 bp 3′-terminal region (3′-UTR), which contains nine ATTTA motifs, and a 4,503 bp open reading frame encoding a polypeptide of 1,500 amino acid residues. Structural analysis indicated that *Bm*Duox contains a typical peroxidase domain at the N-terminus followed by a calcium-binding domain, a ferric-reducing domain, six transmembrane regions and binding domains for flavin adenine dinucleotide (FAD) and nicotinamide adenine dinucleotide (NAD). Transcriptional analysis revealed that *BmDuox* mRNA was expressed more highly in the head, testis and trachea compared to the midgut, hemocyte, Malpighian tube, ovary, fat bodies and silk glands. *BmDuox* mRNA was expressed during all the developmental stages of the silkworm. Subcellular localization revealed that *Bm*Doux was present mainly in the periphery of the cells. Some cytoplasmic staining was detected, with rare signals in the nucleus. Expression of *BmDuox* was induced significantly in the larval midgut upon challenge by *Escherichia coli* and *Bombyx mori* nucleopolyhedrovirus (BmNPV). *BmDuox-*deleted larvae showed a marked increase in microbial proliferation in the midgut after ingestion of fluorescence-labeled bacteria compared to the control. We conclude that reducing *BmDuox* expression greatly increased the bacterial load, suggesting *BmDuox* has an important role in inhibiting microbial proliferation and the maintenance of homeostasis in the silkworm midgut.

## Introduction

Dual oxidases (Duoxes) and NADPH-oxidases (Noxes) have various biological and pathological roles in the generation and regulation of reactive oxygen species (ROS). ROS are by-products of mitochondrial respiration or are produced by activation of Duoxes/Noxes in the immune response to pathogen invasion [1], [2]. ROS is cytotoxic at concentrations above certain thresholds, which can lead to damage of important cellular building blocks, such as DNA, proteins and lipids [3]. This oxidative damage can be counteracted by the action of enzymes, including superoxide dehydrogenase (SOD), glutathione peroxidase (Gpx), catalase and thioredoxin peroxidase, to detoxify ROS by host cells [4]. Increasing evidence suggests cells can achieve homeostasis by production of ROS in response to diverse environmental signals [5]–[14].

Generation of ROS and nitric oxide (NO) was suggested to be a potential bactericidal mechanism in the gut epithelial barrier [15]. *Duox1* and *Duox2* were cloned from human thyroid tissue and highly expressed in mucosal surfaces of the lungs and gastrointestinal tract, respectively [16]–[18]. The participation of epithelial cells as a primary barrier against microbes and their constant encounter with microbes indicate that epithelial *Duox* is involved in ROS-dependent activation of immunity [16], [19]. The *Duox* in the zebrafish *Danio rerio* (*DrDuox*) epidermal cells is involved in paracrine signaling to resolve wound inflammation [20] and *Duox* has been shown to have an antimicrobial function in the zebrafish intestinal epithelium [21]. Noxes/Duoxes are present in vertebrates and have been found also in invertebrates, including *Drosophila melanogaster*, *Anopheles gambiae*, *Aedes aegypti* and *Marsupenaeus japonicus*. The Duox and Nox families are distinguished by the presence of an N-terminal extracellular peroxidase-homology domain (PHD), except for the gp91*^phox^*-like oxidase domain [19], [22]. There is one *Duox* gene and one *Nox* gene in the *Drosophila* genome, and genetic analysis showed infection-induced ROS were not produced in *Duox-*knockout flies but were not influenced in *Nox*-knockout flies. *Duox*-knockout flies failed to control bacteria in the gut and were highly susceptible to gut infection, confirming Duox is the source of infection-induced ROS as a microbicidal effector molecule [8]. In *A. gambiae*, peroxidases and dual oxidase formed a di-tyrosine network between the midgut epithelial and peritrophic membrane that decreased the permeability of the midgut to immune elicitors, protecting the microbiota and malaria parasites in the midgut lumen without activation of epithelial immunity. Pathogen-specific immune responses of the host were activated by disruption of this barrier [23]. The production of Nox in the shrimp *M. japonicas* was considered to be an important factor in innate immunity [24].

Duox/Nox-derived ROS are reported to have important roles in biological and pathological conditions and have been studied extensively in both vertebrates and invertebrates. However, there are few reports for the silkworm *Bombyx mori*, which is an important economic insect and a model organism in the Lepidoptera family, the majority of which are agricultural pests. We undertook characterization of the silkworm dual oxidase gene (*BmDuox*) to help us understand the molecular mechanisms underlying the silkworm immune response. In addition, information obtained for the silkworm will provide leads for the development of more efficient strategies for pest control.

## Materials and Methods

### Materials and Reagents

*B. mori* strain P50 was used for all experiments. Larvae were fed mulberry leaves (*Morus* sp.) and kept at 25±1°C with 70–85% relative humidity and a photoperiod of 12 h light:12 h dark. The RNA extraction kit was from Invitrogen (Carlsbad, CA). The pMD-19 T vector, T4 DNA ligase, restriction enzymes, SMART™ RACE cDNA Amplification Kit and Adavatage® 2 PCR Kit were purchased from TaKaRa (Dalian, China). The pUC-18 T vector, PCR primers and DNA sequencing were acquired from Shanghai Sangon Biological Engineering Technology & Services Co., Ltd.

### Primer Design

A candidate silkworm *BmDuox* gene was identified by TBLASTN analysis using the *D. melanogaster* Dual Oxidase amino acid sequence (GenBank accession number NP_608715). PCR primers used for cloning *BmDuox* are given in Table S1.

### Total RNA Isolation

Total RNA was isolated from various tissues of the 3^rd^ day of the 5^th^ instar larvae and various developmental stages with the Kit (Invitrogen) according to the manufacturer’s instructions and stored at –70°C. The purity of the RNA was determined by the ratio of absorbance at 260 nm and at 280 nm (*A*_260_/*A*_280_) and by agarose (1%) gel electrophoresis.

### Cloning of Full-length *BmDuox* by EST Analysis and 5′/3′ RACE

Two expressed sequence tags (ESTs; GenBank accession numbers CK529174 and CK528168) were found in the silkworm genome database (SilkDB). Full-length *BmDuox* was cloned with RACE-PCR with the SMART™ RACE cDNA Amplification Kit according to the manufacturer’s instructions. Gene-specific primers *BmDuox* RACE-F and RACE-R were designed on the basis of the EST fragment sequence (Table S1). The 5′ first-strand cDNA template was synthesized from silkworm head total RNA (1 µg) in the presence of 5′ RACE CDS primer A and a SMARTer II A Oligonucleotide in a standard reverse transcription buffer containing 100 U/µl of SMARTScribe™ Reverse Transcriptase. The 3′ first-strand cDNA template was obtained from silkworm head total RNA (1 µg) in the presence of 3′ RACE CDS primer A in a similar standard reverse transcription reaction. The Adavatage® 2 PCR Kit was used for RACE-PCR. Amplified products were inserted into the pMD-19 T vector for sequencing.

### Sequence Analysis

The structural domains of *Bm*Duox were predicted using the simple modular architecture research tool (SMART; version 7.0) (http://smart.embl-heidelberg.de/) [25]. During analysis, PFAM domains, signal peptides and internal repeats were checked in the SMART screen page. Translation of the gene and characteristic prediction of the deduced protein were done with ExPASy (http://au.expasy.org). Secretory signal sequences were predicted using SignalP (http://www.cbs.dtu.dk/services/SignalP/). Transmembrane domain searches were done with TMHMM (http://www.cbs.dtu.dk/services/TMHMM-2.0/). Alignments were done with ClustalW (http://www.ebi.ac.uk/Tools/msa/clustalw2/) and MEGA 4 was used to produce a phylogenetic tree (http://www.megasoftware.net/). SilkDB (http://silkworm.genomics.org.cn/silksoft/silkmap.html) was used to predict the chromosomal localization of the *BmDuox* gene. Similarity and identity were analyzed with MatGAT 2.0 [26]. Multiple sequence alignment was done by Florence Corpet (http://multalin.toulouse.inra.fr/multalin/multalin.html) [27]. Tertiary structure was predicted using SWISS-MODEL (http://swissmodel.expasy.org/) [28]–[30].

### Phylogenetic Analysis

The nucleotide and deduced amino acid sequences of *BmDuox* cDNA were analyzed using DNAMAN version 5.2.2. The sequences of Duoxes/Noxes from various species [24] were downloaded from the NCBI database and compared with the NCBI BLAST search program. Multiple sequence alignment was done with ClustalW and the phylogenetic trees of Duoxes/Noxes entire length and oxidase domains from Duoxes were made using the neighbor-joining method in MEGA 4 software. GenBank accession numbers of the Duoxes sequences are given in Table S2.

### Expression Patterns of *BmDuox* mRNA in Various Developmental Stages and Different Tissues of the 3^rd^ day of the 5^th^ Instar Larvae by Real-time Quantitative PCR (RT-qPCR)

RT-qPCR was used to detect and quantify the *BmDuox* gene expression levels in developmental stages and different tissues in the 3^rd^ day of the 5^th^ instar larvae, using the *actin* 3 gene as an internal control. Total RNA was isolated from different developmental stages (from egg to adult). First, the samples were freeze-dried, then a portion of the powder was used for total RNA extraction with the RNA extraction kit according to the user’s manual. Total RNA was isolated from the head, midgut, silk gland, testis, ovary, trachea, fat body, Malpighian tube and hemocyte of the 3^rd^ day of the 5^th^ instar larvae as described above. A 1 µg portion of total RNA was used as the template for the first-strand cDNA synthesis. RT-qPCR was done using SYBR green dye (SYBR Green PCR Master Mix, TaKaRa) with the ABI 7300 System (Applied Biosystems, USA). The *BmDuox* gene expression analysis primers are given in Table S1. The relative expression level of *BmDuox* was determined using the 2^–ΔΔCt^ method [31]. Experiments were done in triplicate for each tissue. Melting curve analysis of amplified products was done at the end of each run to confirm the specificity of amplification.

### Antibody Generation

A partial fragment of *BmDuox* was cloned by RT-PCR (the primers used are given in Table S1). The PCR product was ligated into the pEASY-E1 expression vector. The error-free prokaryotic expression cassette pEASY-E1-*BmDuox* (Figure S1A) was used to transform *E. coli* strain BL21 cells. After induction for 4 h with isopropyl-β-d-thiogalactopyranoside at a final concentration of 1 mmol/L, 1.5 ml of the transformed BL21 cells was harvested for verification of successful induction of the correct antigen, the His-*Bm*Duox fusion protein. The cells were suspended in 100 µl of TE buffer (pH 8.0) and analyzed by sodium dodecyl sulfate/polyacrylamide gel electrophoresis (SDS-PAGE) and western blotting (Figure S1B). Detection used a primary antibody of rabbit anti-6×His (Sigma) and a secondary antibody of horseradish peroxidase (HRP)-conjugated goat anti-rabbit (Sigma).

To purify the recombinant protein, 1 l of BL21 cells was harvested by centrifugation. The pellet was suspended in lysis buffer (10 mM Tris–HCl pH 8.0, 50 mM NaH_2_PO_4_, 100 mM NaCl) and subjected to sonication (5 pulses (200 W), 1 min each) to disrupt the cells. The lysate was centrifuged at 12,000 *g* for 15 min at 4°C, the pellet was discarded and the supernatant was purified by the use of Ni-NTA (Ni^2+^-nitrilotriacetate) resin (Invitrogen, Carlsbad, CA) according to the manufacturer’s instructions. After elution, the concentration of purified protein was determined by the Bradford method using bovine serum albumin (BSA) as the standard. Briefly, antibody was raised in New Zealand rabbits by subcutaneous injection of purified *Bm*Duox (100 mg) expressed in *E. coli* at to me zero (*t*_0_) as described above with an equal volume of Freund’s complete adjuvant and made to a total volume of 1 ml/animal. Booster injections were administered at *t* = 7, 14 and 21 days with the same dose of antigen in Freund’s incomplete adjuvant. Final booster injections were administered at *t* = 28 days with the same dose of antigen in 1 ml of sterile PBS. Serum was collected and stored at –70°C.

The specificity of the antibody was verified by western blotting with extracted total proteins from BmN cells (Figure S1C).

### Subcellular Localization of *Bm*Duox by Immunofluorescence

BmN cells derived from silkworm ovary were grown at 27°C in TC-100 medium supplemented with fetal bovine serum (FBS) at a final concentration of 10% (v/v). BmN cells were fixed in 4% (v/v) paraformaldehyde at room temperature for 2 h and rinsed with 0.01 M PBST (10 mM Na_2_HPO_4_, 1.8 mM KH_2_PO_4_, 140 mM NaCl, 2.7 mM KCl, pH 7.4) containing 0.05% (v/v) Tween-20. The fixed cells were blocked with 3% (w/v) BSA at room temperature for 2 h followed by three washes (5 min each) in PBST then incubated overnight at 4°C with *Bm*Duox polyclonal antibody (diluted 1∶100 in blocking buffer). After three washes (5 min each) in PBST, cells were incubated with anti-rabbit fluorescein isothiocyanate (FITC)-labeled secondary antibody (HuaAn Biotechnology) at a dilution of 1∶200. FITC displays green fluorescence under blue light. The nuclei were labeled with 4′,6-diamidino-2-phenylindole (DAPI), which exhibits blue fluorescence. Cells were examined under a laser confocal scanning microscope (TCS SP5).

### Expression Profiles of *BmDuox in vitro* and *in vivo*

To further confirm the expression profiles of *BmDuox* induced by bacterial challenge, the relative expression levels of *BmDuox in vitro* and *in vivo* were quantified by RT-qPCR. *B. mori* BmN cells were incubated in TC-100 medium supplemented with 10% (v/v) FBS at 27°C. Upon reaching 70–80% confluence, soluble microbial extract (SME) was added to the medium to a final concentration of 10 µg/ml. Cells were collected at 5, 10, 15, 20, 25 and 30 min after challenge. To investigate the *BmDuox* expression pattern *in vivo*, OD_600_ = 1 *E. coli* was spread onto the surface of mulberry leaves, which were then used to feed 5^th^ instar larvae. Midguts were collected at 3, 6, 9, 12 and 24 h after challenge.

### Virus Inoculation

BmNPV was suspended in distilled water to a concentration of 10^6^ polyhedra/ml. A 1 ml sample of viral suspension was spread equally onto 10 pieces of mulberry leaves, each ∼15 cm^2^, which were later fed to 5^th^ instar larvae and control larvae were fed with the same quantity of leaves but treated with distilled water. Midguts were collected at 12, 24, 48, 72 and 96 h after inoculation.

### ROS Detection

The cell-permeant reagent 2′,7′-dichlorodihydrofluorescein diacetate (DCFH-DA) was used as a molecular probe indicator of ROS. After diffusion into the cell, DCFH-DA was deacetylated by cellular esterases to yield the non-fluorescent compound 2′,7′-dichlorofluorescin (DCFH), which was later oxidized by ROS to yield 2′,7′-dichlorofluorescin (DCF). The highly fluorescent DCF can be detected by fluorescence spectroscopy using an excitation wavelength of 488 nm and an emission wavelength of 550 nm [32]. Briefly, *B. mori* BmN cells were challenged with SME (10 µg/ml) for 5, 10, 15, 20, 25 or 30 min, then 1 µl of 10 µM DCFH-DA (0.1 ml of 10 mM DCFH-DA in DMSO; Molecular Probes) was added to each plate and incubated at 27°C for 30 min. Cells were washed three times with double-distilled water (2 ml) and then lysed with 3 ml of double-distilled water. Cell lysates were collected and fluorescence was measured. H_2_O_2_ (final concentration is 2 µM) was added to the cell medium as a positive control.

### Construction of Gene-targeting Vectors and Follow-up Verifications

To construct the gene-targeting vector, the left homologous arm (BmDuox-left), a 1.2 kb fragment, and the right homologous arm (BmDuox-right), a 1.8 kb fragment, were cloned. Reporter gene *egfp* (1.8 kb) was driven with the A3 promoter and the selection gene *neo* (1.7 kb) was driven with the ie-1 promoter from the BmNPV. PCR sequencing was used to confirm the recombinant vectors. All these elements were amplified by PCR (the primers are given in Table S1).

### Production of the Transgenic Silkworm

Sperm-mediated gene transfer (SMGT) was done as described [33], [34]. Briefly, 10 µg of homologous recombination vector was injected into the copulatory pouch of virgin *B. mori* female adults (strain Qiufeng ×Baiyu), which were then free to copulate for 3–4 h. The eggs were maintained until hatching at 25°C in a chamber with 85–90% relative humidity.

### Screening and Identification of the Targeted Silkworm

The expression of enhanced green fluorescent protein (EGFP) in G0 eggs, larvae and pupae was detected using a fluorescence stereomicroscope (SZX12, OLYMPUS). Individuals with GFP-positive G1 offspring were identified as germline-positive transgenic silkworms. Genomes from the G0 generation of the transgenic silkworm moths were monitored by PCR using *egfp*-specific primers *egfp*-1/*egfp*-2 and ie-1-*neo*-sv40polyA-1/ie-1-*neo*-sv40polyA-2.

The genome status of the G0 generation transgenic silkworms was confirmed by dot hybridization with a DIG-labeled ie-1 probe according to the Roche protocols (Roche, DIG High Prime DNA Labeling and Detection Starter Kit I, No. 11745832910).

Midguts from the G0 generation of transgenic silkworm larvae were collected for total protein extraction for western blotting. Briefly, samples were ground into powder and dissolved in lysis buffer (2.5% (w/v) SDS, 10% (v/v) glycerin, 5% (v/v) β-mercaptoethanol and 62.5 mM Tris–HCl pH 6.8) overnight at 4°C. After centrifugation, the supernatant was used for SDS-PAGE and western blotting. The primary antibody used was rabbit anti-GFP (Sigma) and the secondary antibody was HRP-conjugated goat anti-rabbit (Beyotime Institute of Biotechnology).

Positive individuals were used for the extraction of total mRNA and total proteins for analysis of the gene silencing effect. The mRNA of *BmDuox* was identified by RT-PCR, and the mRNA extracted from normal silkworms was used as the control. *Bm*Duox expression was identified by western blotting between normal and positive silkworms.

### *In vivo* Bacterial Persistence Assay

The bacterial persistence assay was done as described [8]. Briefly, spectinomycin-resistant *E. coli-GFP* was coated onto the surface of mulberry leaves and used to feed the 3^rd^ day of the 5^th^ instar larvae. *E. coli-GFP* persistence was measured by plating appropriate dilutions of the homogenates of four surface-sterilized midgut contents and samples were collected at 12 h after infection. The microbes were grown on LB agar plates containing spectinomycin (100 µg/ml) and the number of colony-forming units (CFUs) was counted. The results are expressed as mean and standard deviation of three independent experiments.

### Statistical Analysis

All data are presented as mean ± SD. Statistical differences were evaluated using Student’s *t*-test for unpaired samples. The level of statistically significant difference was set at *P<0.05, **P<0.01 and ***P<0.001.

## Results

### Sequence Analysis of the *BmDuox* Gene

**Figure 1:**
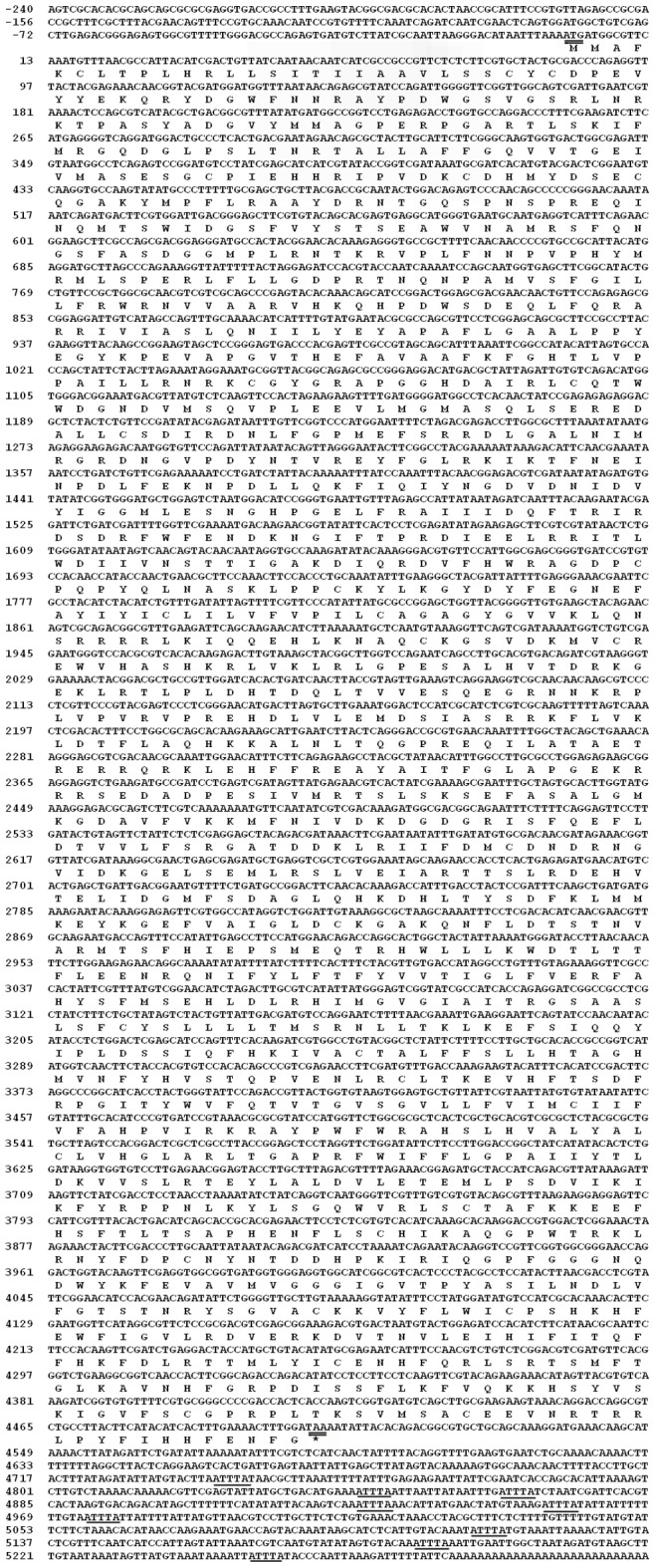
The nucleotide and deduced amino acid sequences of the *B. mori* dual oxidase (*BmDuox*) cDNA. The cDNA (5,545 bp) contains a complete ORF encoding a protein of 1,500 amino acid residues (residue number indicated on the left). The sequence has been deposited in the GenBank (accession number JQ768349). The start codon ATG and the stop codon TAA are indicated by double underscoring. Polyadenylation ATTTA is indicated with a single line.

The full length of *BmDuox* cDNA was obtained using RACE (Figure S2) and bioinformatics approaches. The *BmDuox* cDNA was 5,545 bp long with a 4,503 bp open reading frame (ORF) coding for 1,500 amino acids. It contained a 28 amino acid residue signal peptide at the N-terminus, a 240 bp 5′-untranslated region (5′-UTR) and an 802 bp 3′-untranslated region (3′-UTR). The signal for polyadenylation, ATTTA, was found at the 3′-UTR (Figure 1). The deduced amino acid sequence had a predicted molecular mass of 172 kDa and an isoelectric point of 8.96. This cDNA sequence was deposited with GenBank (accession number JQ768349). Six transmembrane domains were identified in *Bm*Duox using TMHMM online software (Figure S3A). A search of SilkDB revealed that *BmDuox* is localized on the 8th chromosome (Figure S3B).

Blasting the *BmDuox* cDNA against the NCBI *B. mori* genome database revealed it was distributed on genomic DNA fragments AADK01028014.1, AADK01003202.1, AADK01000925.1 and AADK01010320.1. The *BmDuox* cDNA was aligned with the silkworm genomic sequence, and sites for exon–intron borders were deduced by comparison. The *BmDuox* gene was organized into 26 exons and 25 introns. Further comparison with its orthologs from other organisms revealed a generally well-conserved exon–intron organization. All exon–intron splicing junctions matched canonical consensus sequences for donor and acceptor sites (data not shown). Exon 1 contained the start codon, exon 26 contained the termination codon and the 3′-UTR harbored a polyadenylation signal.

### Domain Structure

**Figure 2:**
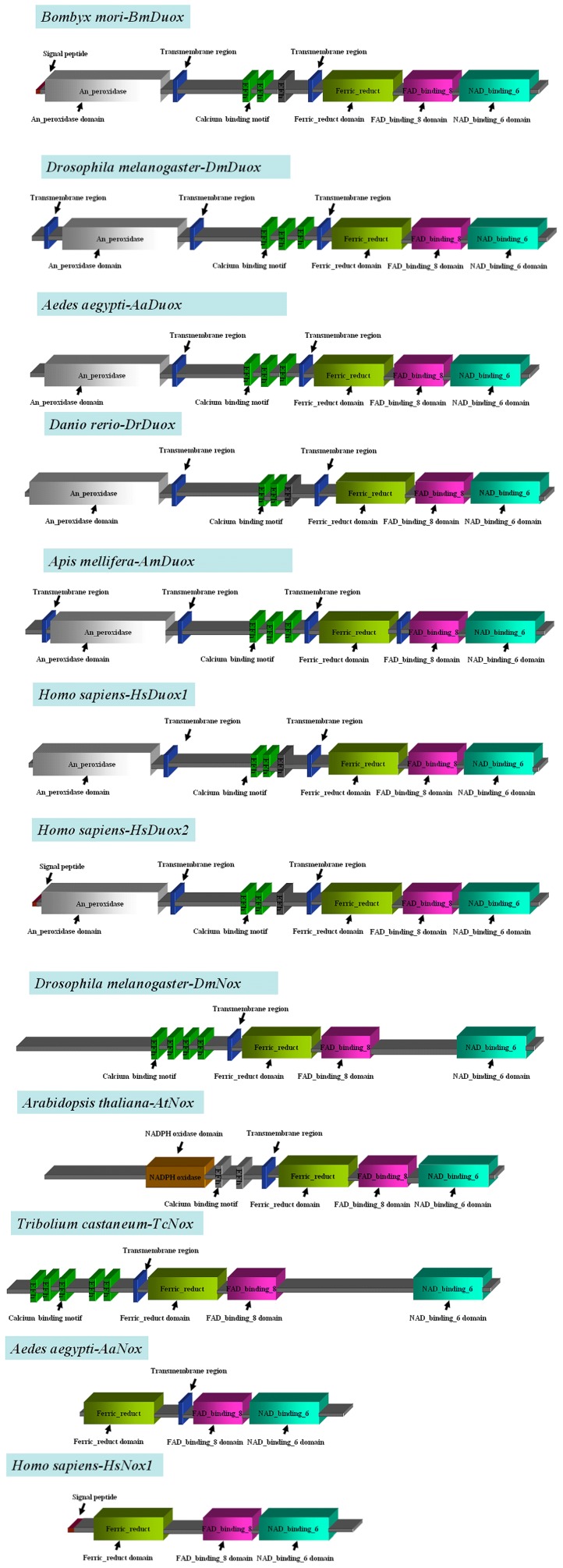
Domain organizations of Duox/Nox proteins. The calcium-binding region contains EF-hand motifs in *Bm*Duox. The ORFs from *Bm*Duox and Duox/Nox of other species are compared: *Bombyx mori* Duox, *Bm*Duox (JQ768349); *Drosophila melanogaster* Duox, *Dm*Duox (NP_608715.2); *Aedes aegypti* Duox, *Aa*Duox1 (XP_001658452.1); *Danio rerio* Duox, *Dr*Duox (BAF33370.1); *Homo sapiens* Duox2, *Hs*Duox2 (NP_054799.4); *Homo sapiens* Duox1, *Hs*Duox1 (NP_059130.2); *Drosophila melanogaster* Nox, *Dm*Nox (NP_001097336); *Arabidopsis thaliana* Nox, *At*Nox (O81211.2); *Tribolium castaneum* Nox, *Tc*Nox (XP_972375.2); *Aedes aegypti* Nox, *Aa*Nox (EAT37894); *Homo sapiens* Nox1, *Hs*Nox1 (CAI42337.1).

The complete domain structures of *Bm*Duox were compared to other Duoxes/Noxes by the SMART program (Figure S3C). The protein contained six transmembrane regions (at positions 593–615, 887–992, 1027–1049, 1080–1102, 1132–1154 and 1175–1197) and binding domains for FAD (at positions 1218–1319) and NAD (at positions 1325–1481) known to be functionally important in various electron transport systems. The arrangement of these domains was found to be common to Duox and Nox proteins (Figure 2). In addition to these domains, *Bm*Duox harbored a peroxidase domain (at positions 36–556), a ferric–reductase domain (at positions 1035–1183) and an N-terminal calcium-binding domain containing 3 EF-hand motifs (at positions 822–850, 858–886 and 903–931), which was found in Duoxes/Noxes of many species; e.g. *D. melanogaster Dm*Duox, *D. rerio Dr*Duox, *Arabidopsis thaliana At*Duox and *Homo sapiens* Duox2/1). The deduced *Bm*Duox protein sequence had a signal peptide of 28 amino acid residues in the N-terminus with an excision site located at Cys28/Asp29. Seven tertiary structure models were predicted using SWISS-MODEL (Figure S4). Compared to *Bm*Duox, *Dm*Duox and *Dr*Duox, the structure domains showed conserved similarity in these tertiary models (data not shown).

### Sequence Homology

The *Bm*Duox amino acid sequence was aligned with published sequences of Duoxes of other species (Figure S5). *Bm*Duox contained a peroxidase domain, a calcium-binding domain, six transmembrane regions, a ferric-reductase domain, an FAD-binding domain and an NAD-binding domain with 521, 87, 43, 149, 102 and 157 amino acid residues, respectively. Sequence alignment was used to determine percentage homology with other Duoxes. *Bm*Duox shared 72.6% sequence homology with *Dm*Duox, 75% with *Aa*Duox1, 71.7% with *Am*Duox and more than 37% with other species Duoxes (Table 1). The *Bm*Duox peroxidase domain shared 67.3% sequence homology with *Dm*Duox, 62.3% with *Am*Duox and 68.6% with *Aa*Duox1 (Table S3A). Duoxes/Noxes of all species contained the transmembrane region and the FAD-binding domain (Figure 2). For the transmembrane region, *Bm*Duox shared 57.3% homology with *Dm*Duox and 71.4% with *Aa*Duox1 but only 24.1% with *Dr*Duox (Table S3B). For the calcium-binding motif, *Bm*Duox shared 89.7% homology with *Dm*Duox, 88.5% with *Am*Duox and 89.7% with *Aa*Duox1 (Table S3C). The *Bm*Duox ferric-reductase domain shared 77.9% homology with *Dm*Duox, 76.5% with *Am*Duox and 83.2% with *Aa*Duox (Table S3D). For the *Bm*Duox FAD-binding domain, there was 86.3% sequence homology with *Dm*Duox, 47.1% with *Dr*Duox, 88.3% with *Am*Duox and 88.2% with *Aa*Duox1 (Table S3E). Finally, the *Bm*Duox NAD-binding domain shared 96.2% homology with *Dm*Duox, 93% with *Am*Duox and 96.8% with *Aa*Duox (Table S3F). The amino acid sequences (SGQWVR, FTLTSAPHEN and GIGVTPYAS) in the NAD-binding domains of vertebrates and invertebrates were all conserved (Figure S6). These results showed that *Bm*Duox has a high level of amino acid sequence homology with Duoxes from various species. The analysis of individual domain homology among various species revealed high levels of similarity and conservation.

**Table 1:** Amino acid identity and similarity of the silkworm *BmDuox* gene compared to other known Duoxes/Noxes sequences using the entire Duoxes/Noxes protein.

Full-length	1	2	3	4	5	6	7	8	9	10	11	12	13
1. *Bm*Duox		37.3	72.6	38.3	39.2	38.8	38.9	39.1	38.8	38.0	37.7	71.7	75.0
2. *Dr*Duox	62.0		36.9	54.1	55.8	55.0	55.7	54.9	55.3	55.8	55.5	37.9	38.1
3. *Dm*Duox	84.6	60.9		37.4	38.9	38.6	38.3	38.9	38.1	38.0	37.6	72.4	83.1
4. *Hs*Duox2	60.2	71.6	60.0		77.7	82.6	74.8	84.0	74.7	87.5	86.9	38.7	38.3
5. *Hs*Duox1	60.5	73.1	59.9	86.9		73.8	90.2	74.4	91.0	75.4	75.2	38.9	39.1
6. *Rn*Duox2	61.0	72.2	61.5	90.5	84.1		75.5	94.1	74.6	80.9	80.5	38.6	39.2
7. *Rn*Duox1	60.7	72.9	60.1	85.2	94.7	85.0		75.4	96.4	75.0	74.4	38.6	38.6
8. *Mm*Duox2	61.6	72.6	61.9	91.6	84.5	97.2	84.8		76.0	81.8	81.4	39.4	39.2
9. *Mm*Duox1	60.9	73.0	60.1	85.1	95.2	84.8	98.2	84.9		75.1	74.9	38.2	38.3
10. *Bt*Duox2	60.0	72.6	60.6	92.6	85.2	89.6	85.5	89.9	85.8		97.1	38.6	38.6
11. *Oa*Duox2	59.7	71.9	60.1	92.1	84.7	89.1	84.8	89.3	85.0	98.2		38.3	38.3
12. *Am*Duox	85.0	61.7	83.8	60.7	60.4	61.7	60.7	62.2	60.3	61.0	60.8		75.1
13. *Aa*Duox1	86.8	61.5	90.4	59.9	59.5	61.0	59.8	61.4	59.9	60.0	59.5	88.3	

Upper triangle: identity, lower triangle: similarity.

### Phylogenetic Analysis

**Figure 3:**
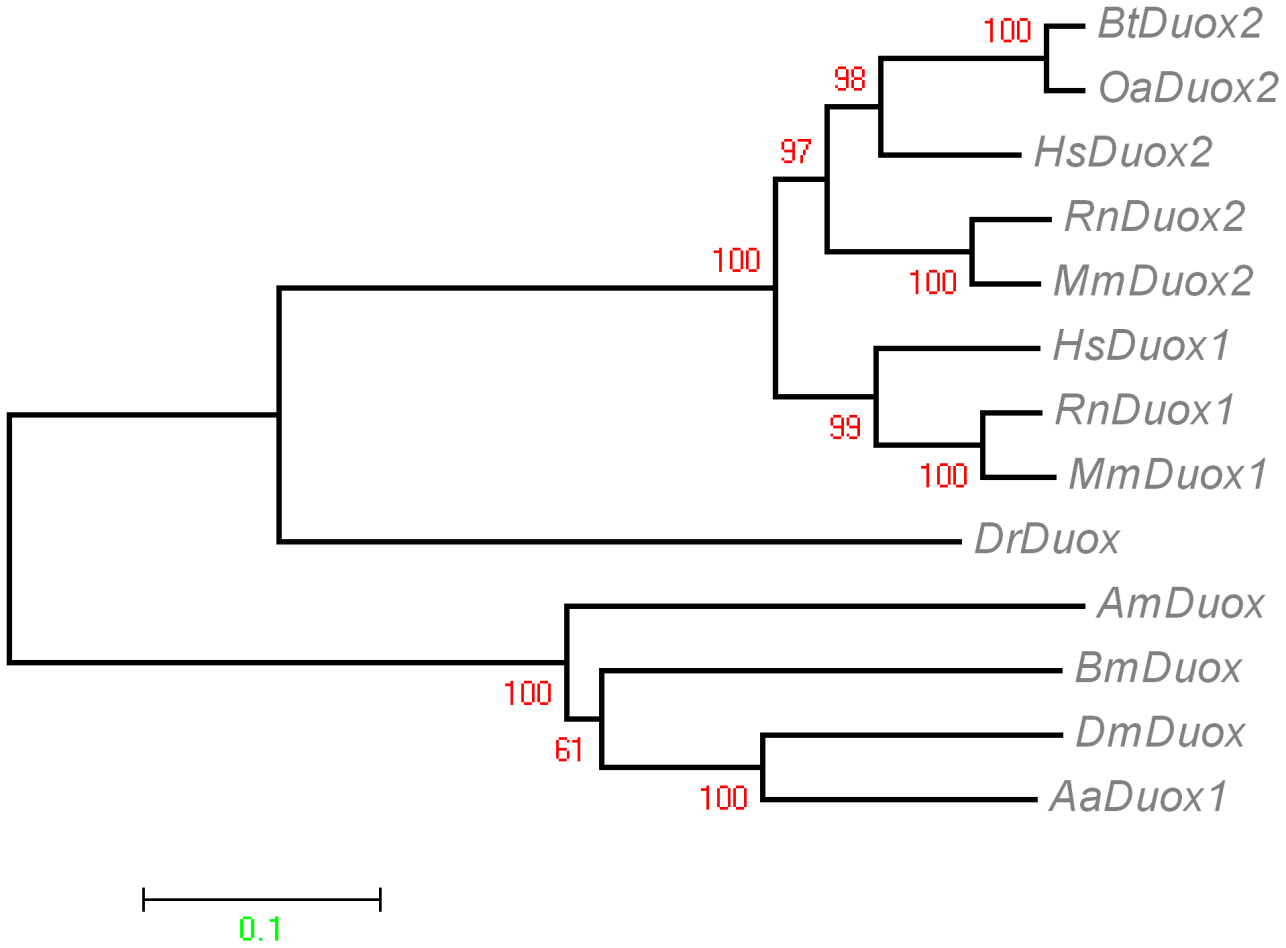
Phylogenetic trees of the relationship between *Bm*Duox and Duoxes from homologous species were constructed using the neighbor-joining program with bootstrapping using 1000 replicates. The bootstrap values are shown on each internal node. The deduced oxidase domain sequences of Duoxes were analyzed using ClustalW and MEGA 4 software. GenBank accession numbers of the other Duoxes sequences are given in Table S2.

To better elucidate the evolutionary relationships between *Bm*Duox and other sequenced Duoxes proteins, a molecular taxonomy was constructed by aligning the oxidase domains of the *Bm*Duox and other species of Duoxes. The accession numbers, symbols and nomenclature used in the phylogenetic analysis of sequences are given in Table S2. *Bm*Duox formed a cluster with insect Duoxes, including those of *D. melanogaster*, *Apis mellifera* and *A. aegypti* (Figure 3). *D. rerio Dr*Duox and *H. sapiens Hs*Duox2 formed another cluster. The whole sequence of *Bm*Duox and other species of Duoxes/Noxes was used to determine the evolutionary relationships (Figure S7).

### Expression Profile of *BmDuox* in various Developmental Stages and Different Tissues by RT-qPCR

The *BmDuox* gene was expressed throughout all four developmental stages of the silkworm; namely, egg, larva, pupa and adult (Figure S8A). Furthermore, analysis of the tissue distribution of the *BmDuox* gene showed it was highly expressed in the head, testis and trachea, with low levels of expression detected in the midgut, hemocyte, Malpighian tube, ovary, fat bodies and silk glands (Figure S8B).

### Subcellular Localization of *Bm*Duox

*Bm*Duox polyclonal antibody and FITC-labeled goat anti-rabbit IgG were used as primary and secondary antibodies, respectively, to examine the localization of *Bm*Duox. Cells were examined under a laser confocal scanning microscope (TCS SP5). DAPI-stained nuclei emit blue fluorescence upon stimulation by UV. Our results indicated that *Bm*Doux was present mainly in the periphery of the cells. Some cytoplasmic staining was detected, with rare signals in the nucleus, suggesting this protein is localized in the cell membrane (Figure S9).

### *BmDuox* Induction, Expression Analysis and Generation of ROS

To further explore the possible *BmDuox* expression pathways at the molecular level, BmN cells were treated with SME and the mRNA expression of *BmDuox* was later examined. Our results showed the level of ROS in BmN cells was increased significantly by SME (Figure S10A). In addition, the initial level of *BmDuox* mRNA expression was basal and was later induced by SME (Figure S10B). The expression pattern of *BmDuox* upon challenge by bacteria (DH5α, Gram-negative bacteria) (Figure S10C) and virus (BmNPV) (Figure S10D) *in vivo* was examined and the results showed expression of *BmDuox* was induced at specific time points in the silkworm midgut.

### Screening and Identification of Transgenic Silkworms

**Figure 4:**
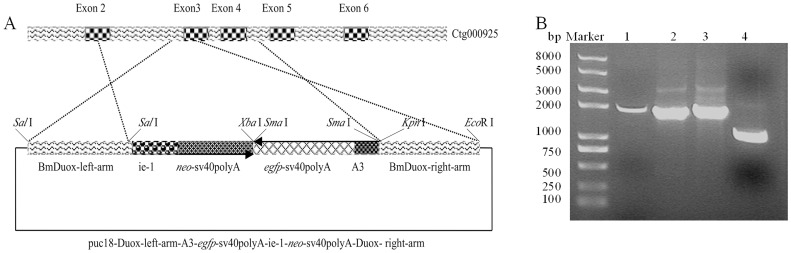
The structure of homologous recombination vectors. (A) puc18-Duox-left-arm-A3-*egfp*-sv40polyA-ie-1-*neo*-sv40polyA-Duox-right-arm. *gfp*, green fluorescent protein gene; A3, cytoplasmic actin A3 gene promoter; sv40polyA, simian vacuolating virus 40 poly(A) signal; *ie*-*1*, baculovirus *ie*-1 promoter; *neo*, neomycin resistance gene. (B) Marker, DNA ladder (100–8,000 bp); 1, BmDuox-left-arm; 2, ie-1-*neo*-sv40polyA; 3, A3-*egfp*-sv40polyA; 4, BmDuox-right-arm.

**Figure 5:**
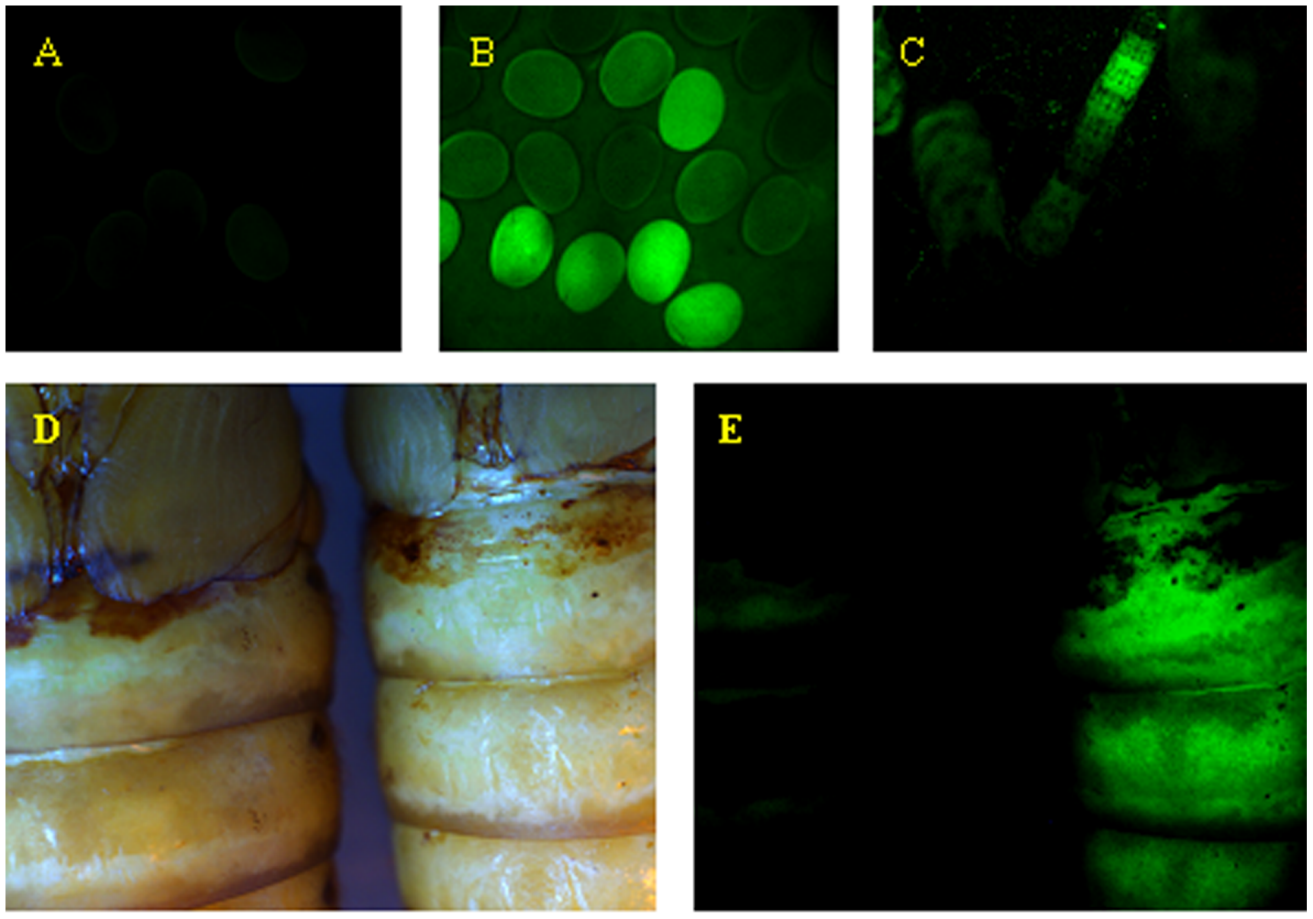
Identification of *BmDuox* knockout silkworms by fluorescence microscopy. (A) Egg laid by non-transgenic silkworm (B) Fluorescence of eggs laid by G1 generation of the *BmDuox* knockout silkworm. (C) Third instar larva of the G1 generation of *BmDuox* knockout silkworm. (D) Normal pupa (left) and targeted pupa (right) under natural light. (E) Fluorescent pupa of the G1 generation of *BmDuox* knockout normal (left) and targeted pupa (right).

To investigate the role of the *BmDuox* gene *in vivo*, we generated a knockout silkworm with the *BmDuox* gene silenced by transgenic methods. The *BmDuox* gene was disrupted by insertional mutagenesis. A vector containing *A3*-*egfp-sv40polyA* and *ie1*-*neo*-*sv40polyA* expression cassettes was integrated into the silkworm genome through homologous recombination (Figure 4). Fluorescence was detected in fertilized eggs (Figure 5A and 5B), 3^rd^ instar larvae (Figure 5C) and pupae (Figure 5D and 5E), demonstrating the *egfp* gene was expressed continuously *in vivo*.

**Figure 6:**
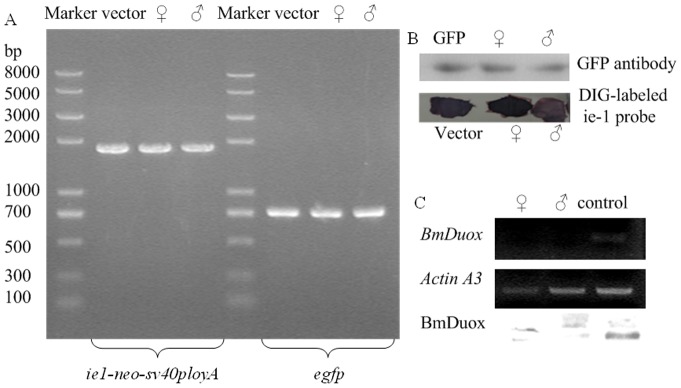
Identification of transgenic silkworms by molecular biology methods. (A) Amplification with ie-1-*neo*-sv40polyA-1/ie-1-*neo*-sv40polyA-2 primers (left) and *egfp*-1/*egfp*-2 primers (right) from fluorescent silkworms. (B) Identification of fluorescent silkworms with western blotting using GFP antibody (up) and by dot hybridization with a DIG-labeled ie-1 probe (down). (C) RT-PCR (up) and western blotting (down) analysis of the knockout silkworms (fluorescent silkworms) and normal silkworms.

Disruption of the *BmDuox* gene was verified using PCR analysis of genomic DNA extracted from transgenic silkworms that were positive for *egfp* fluorescence. Specific bands of the *ie1*-*neo*-*sv40polyA* (∼1800 bp) expression cassette and the *egfp* gene (∼720 bp) were amplified (Figure 6A). All the PCR products amplified from G1 transgenic silkworms were recovered from agarose gel after separation by electrophoresis and further verified by DNA sequencing (data not shown). In addition, the foreign protein expression was verified by western blotting analysis of total proteins extracted from transgenic silkworms that were positive for *egfp* fluorescence. An expected band of ∼26 kDa was identified in fluorescent silkworms with the GFP-labeled antibody (Figure 6B). Dot hybridization with an *ie*-*1 *gene probe, which was used to verify the presence of the foreign gene fragment in fluorescent silkworms, showed the genomes of fluorescent moths of generation G1 contained the *ie*-*1 *gene (Figure 6B). Endogenous gene expression was verified by RT-PCR and western blotting analysis of mRNA and total proteins extracted from transgenic silkworms positive for *egfp* fluorescence. The levels of expression of *BmDuox* mRNA and *Bm*Duox protein were reduced significantly compared to control silkworms (Figure 6C).

### *In vivo* Bacterial Persistence Assay

**Figure 7:**
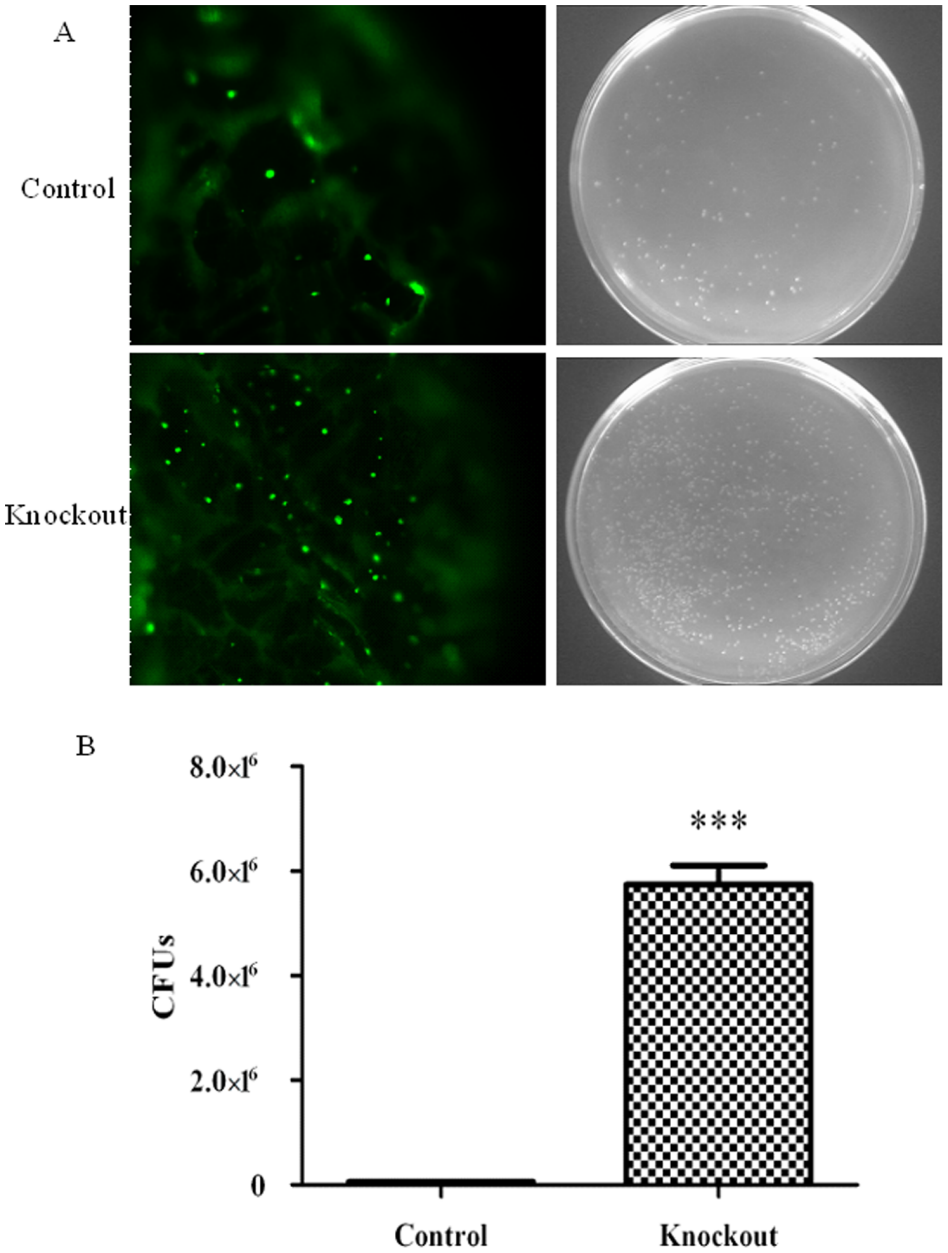
Persistence of *E. coli-GFP* in the peritrophic membrane of *BmDuox* knockout and normal silkworms. (A) Persistence of *E. coli-GFP* (spectinomycin-resistant) in the peritrophic membrane of normal silkworm and representative plates of *E. coli-GFP* in the peritrophic membrane (up); persistence of *E. coli-GFP* (spectinomycin-resistant) in the peritrophic membrane of *BmDuox* knockout silkworm and representative plates of *E. coli-GFP* in the peritrophic membrane (down). (B) Knockdown of *BmDuox* reduces the capacity of larvae to clear *E. coli-GFP* in the midgut. The graph represents relative numbers of *E. coli-GFP* CFUs. Results are expressed as the mean and standard deviation of three different experiments. Statistical differences were evaluated using Student’s *t*-test for unpaired samples; *P<0.05, **P<0.01, ***P<0.001.

To investigate the relationships between *BmDuox* activity and microbial persistence *in vivo*, we examined the midgut of *BmDuox*-silenced larvae during natural infection, using the GFP-tagged pathogen *E. coli-GFP*. Dramatic differences in midgut loads were observed between wild type (wt) and *BmDuox* knockdown (kd) silkworms. *BmDuox* kd larvae harbored 100-fold more CFUs compared to wt silkworm controls 12 h after bacterial feeding (Figure 7).

## Discussion

The roles of ROS from Duox and Nox have been studied extensively in vertebrates and invertebrates but the role of ROS released during activation of the *BmDuox* gene in the silkworm, an important economic insect and a model organism in the Lepidoptera family, is not known. The *BmDuox* gene is expected to be of great importance in the response of silkworm innate immunity upon challenge by pathogens. In this study, the full length of the *BmDuox* gene was cloned. The ORF is 4,503 bp long and encodes a 1,500 amino acid protein with a theoretical mass of 172 kDa, which is to *M. japonicus* (∼173 kDa), *D. rerio* (∼172 kDa) and *D. melanogaster* (∼178 kDa). The amino acid sequence of *Bm*Duox has a high level of identity with Duoxes and some Noxes of other species, indicating Duoxes/Noxes are highly conserved among species. Furthermore, the signal peptide in the N-terminus was found only in *Bm*Duox and *Hs*Duox2.

Analysis of the *Bm*Duox amino acid sequence revealed a peroxidase domain and a calcium-binding domain at the N-terminus and conserved FAD- and NAD-binding domains at the C-terminus. The structure of *Bm*Duox was similar compared to Duoxes from invertebrates [35], [36] and vertebrates [37], [38], [39]. There is a high level of identity among Duoxes, suggesting the role of these domains is conservative and *Bm*Duox might have multi-functions that distinguish it from other organisms [40].

The peroxidase domain present in the N-terminus of Duoxes from various species is absent from Noxes. *Dm*Duox has an additional peroxidase domain that could produce ROS in a regulated manner [41]. Although tissues are likely to be damaged by excessive ROS, over-proliferation of intestinal microbes is limited, indicating the role of ROS *in vivo* is controlled mainly by the interactions of multiple genes [8].

N-terminal EF-hand motifs are present in the calcium-binding domain. However, except for human *Hs*Nox1 and *A. aegypti Aa*Nox, a calcium-binding domain was found in all the afore-mentioned Duoxes/Noxes. The most diverse characteristic of the calcium-binding domain among various species is the number of EF-hand motifs. The *Bm*Duox, *Dm*Duox, *Hs*Duox and *Dr*Duox calcium-binding domains include three EF-hand motifs. It has been shown that intracellular concentrations of Ca^2+^ can moderate *Drosophila* Duox enzymatic activity via the EF-hand motifs [15]. Human Duox1 and Duox2 have a calcium-binding domain and Ca^2+^ is considered a regulatory factor for the activation of Duox via a pair of EF-hands [9]. Therefore, we suggest that the EF-hands of *Bm*Duox are involved in the response to Ca^2+^ in a manner similar to those of the *Drosophila* and human Duoxes.

The transmembrane region of *Bm*Duox contains six transmembrane sites, whereas *Dm*Duox and *Am*Duox possess seven such sites. *Bm*Duox has three phenylalanine and four glycine residues in transmembrane sites that are well conserved among the afore-mentioned Duoxes. The role of these conserved sites in the transmembrane sites warrants further study.

Homology analysis showed both the FAD- and NAD-binding domains of *Bm*Duox share similar homologies with other species. The amino acid sequences SGQWVR, FTLTSAPHEN and GIGVTPYAS in the NAD-binding domains of vertebrates and invertebrates are all conserved. The NAD-binding site has an important role in transporting electrons from NADPH to FAD in *Hs*Nox2 [13]. Therefore, the presence of these conserved sites in the NAD-binding domain of *Bm*Duox suggests a crucial role for this protein in the electron delivery system.

In an *in vitro* time-course (5, 10, 15, 20, 25 and 30 min) experiment, the *BmDuox* mRNA expression level was initially basal, and was induced after stimulation with SME with a peak at 25 min. Similarly, expression of *HsNox2* in human phagocytes was activated by stimulation with lipopolysaccharide (LPS) and interferon-γ (IFN-γ) [42]. The trend of ROS production in BmN cells is consistent with the *BmDuox* mRNA expression level, which was increased at 48 h after viral stimulation followed by a sudden decrease. We suggest the increased level of *BmDuox* mRNA expression in the early stage of stimulation could be due to the immune response of host cells. The expression of *BmDuox* decreased in the early stage of stimulation, indicating pathogenic stimuli can inhibit the expression of *BmDuox* mRNA in host cells. Similar results have been reported for the mud crab [40] and the Chinese shrimp [43]. Our results suggest *BmDuox* is involved in the production of ROS and the immunoprotective response.

We established a sperm-mediated homologous recombination technique and achieved a silkworm site-specific knockout mutant. Microbial persistence and proliferation within the midgut of the *BmDuox*-silenced larvae was much more severe compared to the control group. Dramatic differences in midgut loads were observed between wt and *BmDuox* knockdown (kd) silkworms after bacterial feeding, suggesting *BmDuox* has an important biological defense factor preventing pathogen infection because *BmDuox* mRNA was highly expressed after pathogenic stimuli. On the contrary, down-regulation of *BmDuox* mRNA expression would lead to increased bacterial load in the midgut. In *D. melanogaster*, Duox has an important role in host–microbe homeostasis [8], [44]. *Dr*Duox was shown to have an antimicrobial function in the intestinal epithelium [21]. A peroxidase/Duox system in *A. gambiae* was used to protect microbiota and malaria parasites in the midgut microbiota by preventing the activation of epithelial immunity to release immune elicitors [23].

We conclude the silkworm *BmDuox* gene has an important role in the innate immunity system. More studies are needed to fully understand the mechanism underlying the action of the *BmDuox* gene in innate immunity at the molecular level.

## Supporting Information

Figure S1 **Expression** ***Bm*** **Duox in** ***E. coli*** **,** ***Bm*** **Duox antibody preparation from New Zealand rabbits and the specificity of polyclonal antibody detection using extracted BmN cells.** (A) Identification of pEASY-E1-*BmDuox* with *Bam*H I and *Hin*d III enzymes. (B) *Bm*Duox samples were resolved by SDS/12% polyacrylamide gel electrophoresis (SDS-PAGE) under normal conditions. Left, SDS-PAGE; 3, protein molecular mass marker; 4, recombinant *Bm*Duox expression in *E. coli*. Right, western blotting; 5, protein molecular mass marker; 6, recombinant *Bm*Duox expression in *E. coli*. (C) The specificity of the antibody was verified by western blotting with extracted BmN cells. 7 and 8, *Bm*Duox protein band; 9, protein molecular mass marker.
(TIF)

Figure S2 **PCR analysis.** (A) The 3′ RACE product of ∼1,000 bp (containing the C-terminal partial CDS sequence). (B) The 5′ RACE product of ∼1300 bp (containing the N-terminal partial CDS sequence); M, Trans2K DNA marker.
(TIF)

Figure S3 **Transmembrane region analysis and chromosomal localization of** ***BmDuox*** **.** (A) Six transmembrane regions in *Bm*Duox. (B) Chromosomal localization of *BmDuox*, three red-shaded stars indicate the best hit. (C) Predicted domain organizations of *Bm*Duox from the silkworm.
(TIF)

Figure S4 **Tertiary structure of** ***Bm*** **Duox predicted by SWISS-MODEL.**
(TIF)

Figure S5 **Comparison of the deduced amino acid sequence of NADPH oxidase (** ***Bm*** **Duox) from** ***B. mori*** **with other Duoxes.** *Bm*Duox was aligned with Duoxes from other species; *Bm*Duox (JQ768349), *Dm*Duox (NP_608715.2), *Dr*Duox (BAF33370.1), *Hs*Duox2 (NP_054799.4), *Hs*Duox1 (NP_059130.2), *Rn*Duox2 (NP_077055.1), *Rn*Duox1 (NP_714961.1), *Mm*Duox2 (NP_808278.2), *Mm*Duox1 (NP_001092767.1), *Bt*Duox2 (XP_002690988.1), *Oa*Duox2 (NP_001177321.1), *Am*Duox (XP_624355.3) and *Aa*Duox1 (XP_001658452.1) using the online multiple sequence alignment tool (http://multalin.toulouse.inra.fr/multalin/multalin.html). Red, high consensus; blue, low consensus; black, neutral consensus. Consensus levels: high, 90%; low, 50% must be less than the first value.
(TIF)

Figure S6 **Comparison of NAD-binding domain amino acid sequence of** ***Bm*** **Duox with Duoxes of other species using the online multiple sequence alignment tool (** http://multalin.toulouse.inra.fr/multalin/multalin.html **).** Red, high consensus; blue, low consensus; black, neutral consensus. Consensus levels: high, 90%; low, 50% must be less than the first value.
(TIF)

Figure S7 **Phylogenetic relationship of the Duoxes and Noxes from the silkworm, other insects, vertebrates and plants.** The deduced amino acid sequences were analyzed with ClustalW and MEGA 4 software. GenBank accession numbers of the other Duoxes/Noxes sequences are given in Table S2.
(TIF)

Figure S8 **RT-PCR analysis of** ***BmDuox*** **expression in different developmental stages and different tissues.** (A) Spatio-temporal distribution of *BmDuox* mRNA from egg to adult. (B) Tissue distribution of *BmDuox* mRNA in 3^rd^ day of 5^th^ instar larvae. The *actin* 3 gene was used as the internal control.
(TIF)

Figure S9 **Subcellular localization of** ***Bm*** **Duox in BmN cells by immunofluorescence.** BmN cells were fixed, probed with rabbit polyclonal *Bm*Duox antibody to detect *Bm*Duox, and visualized by FITC-labeled goat anti-rabbit IgG (green). Additionally, cells were stained with DAPI to visualize nuclear DNA (blue) directly. Cells were examined under a laser confocal scanning microscope (TCS SP5).
(TIF)

Figure S10 **Induction expression of** ***BmDuox*** **and ROS generation in BmN cells and midgut.** (A) ROS generation after SME treatment in BmN cells. (B) *BmDuox* expression pattern after SME treatment in BmN cells. (C) *BmDuox* expression pattern after DH5α treatment in the midgut. (D) *BmDuox* expression pattern after BmNPV treatment in the midgut. Results are expressed as the mean and standard deviation of three different experiments. Statistical differences were evaluated using Student’s *t*-test for unpaired samples. *P<0.05, **P<0.01, ***P<0.001.
(TIF)

Table S1 **PCR primers used for** ***BmDuox*** **gene analysis.**
(DOC)

Table S2 **The symbols, GenBank accession numbers and nomenclature used in the phylogenetic analysis.**
(DOC)

Table S3 **Amino acid identity and similarity of the silkworm** ***Bm*** **Duox gene compared to other known Noxes/Duoxes sequences using each domain.** Upper triangle, identity; lower triangle, similarity. A, Peroxidase domain; B, transmembrane region; C, calcium-binding region; D, ferric-reductase; E, FAD-binding domain; F, NAD-binding domain.
(DOC)
