# Supplementary figures and images for: Molecular Cloning and Functional Characterization of the Dual Oxidase (BmDuox) Gene from the Silkworm Bombyx mori
Source: PLoS One. 2013 Aug 2;8(8):e70118. doi: 10.1371/journal.pone.0070118 (PMC3732266; doi:10.1371/journal.pone.0070118)

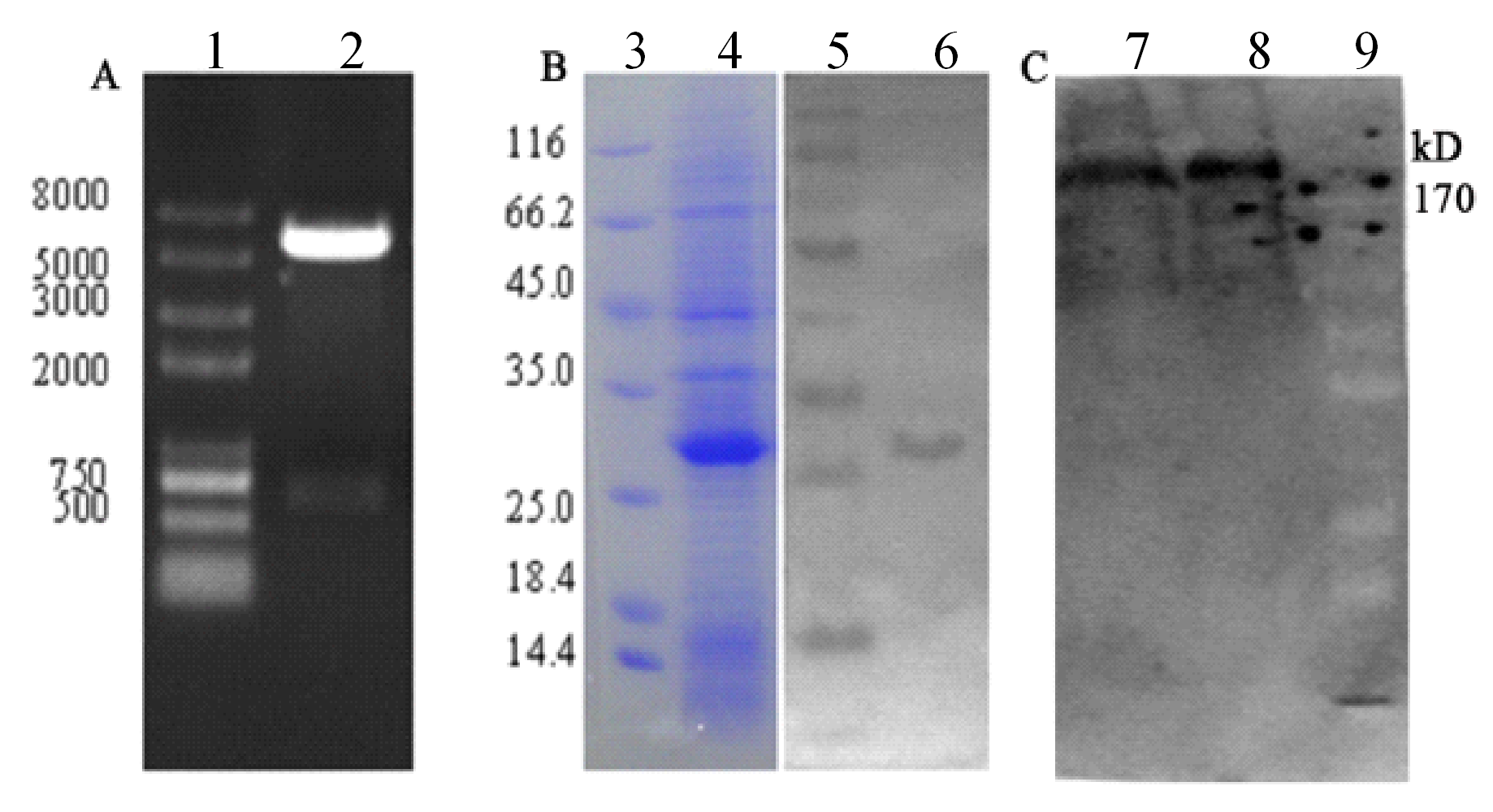

Supplement: Figure S1 — Expression Bm Duox in E. coli , Bm Duox antibody preparation from New Zealand rabbits and the specificity of polyclonal antibody detection using extracted BmN cells. (A) Identification of pEASY-E1-BmDuox with BamH I and Hind III enzymes. (B) BmDuox samples were resolved by SDS/12% polyacrylamide gel electrophoresis (SDS-PAGE) under normal conditions. Left, SDS-PAGE; 3, protein molecular mass marker; 4, recombinant BmDuox expression in E. coli. Right, western blotting; 5, protein molecular mass marker; 6, recombinant BmDuox expression in E. coli. (C) The specificity of the antibody was verified by western blotting with extracted BmN cells. 7 and 8, BmDuox protein band; 9, protein molecular mass marker. (TIF) [file pone.0070118.s001.tif]

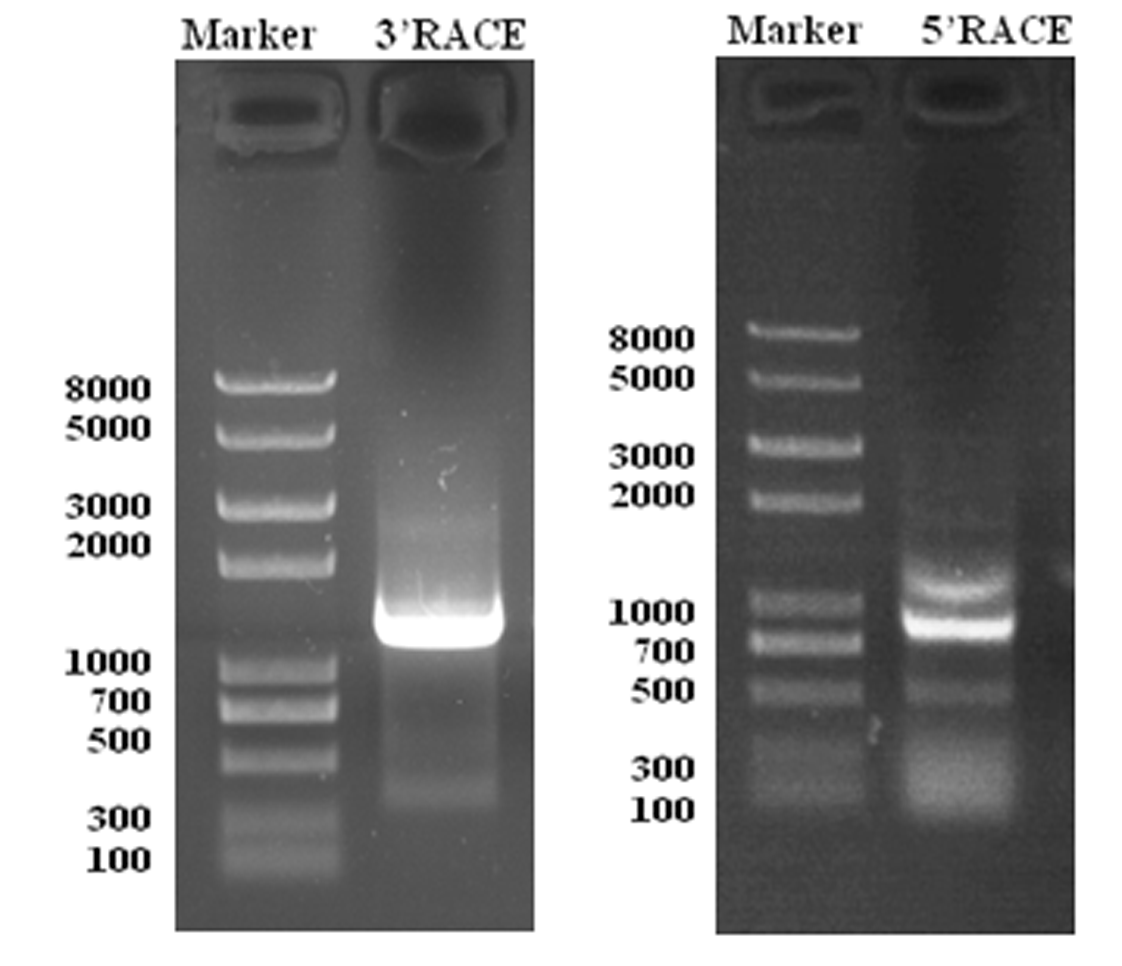

Supplement: Figure S2 — PCR analysis. (A) The 3′ RACE product of ∼1,000 bp (containing the C-terminal partial CDS sequence). (B) The 5′ RACE product of ∼1300 bp (containing the N-terminal partial CDS sequence); M, Trans2K DNA marker. (TIF) [file pone.0070118.s002.tif]

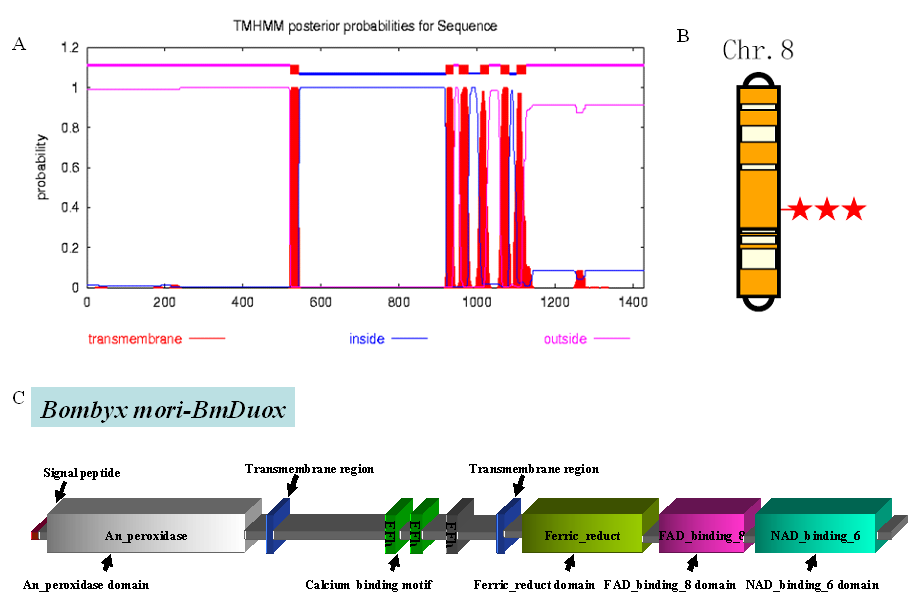

Supplement: Figure S3 — Transmembrane region analysis and chromosomal localization of BmDuox . (A) Six transmembrane regions in BmDuox. (B) Chromosomal localization of BmDuox, three red-shaded stars indicate the best hit. (C) Predicted domain organizations of BmDuox from the silkworm. (TIF) [file pone.0070118.s003.tif]

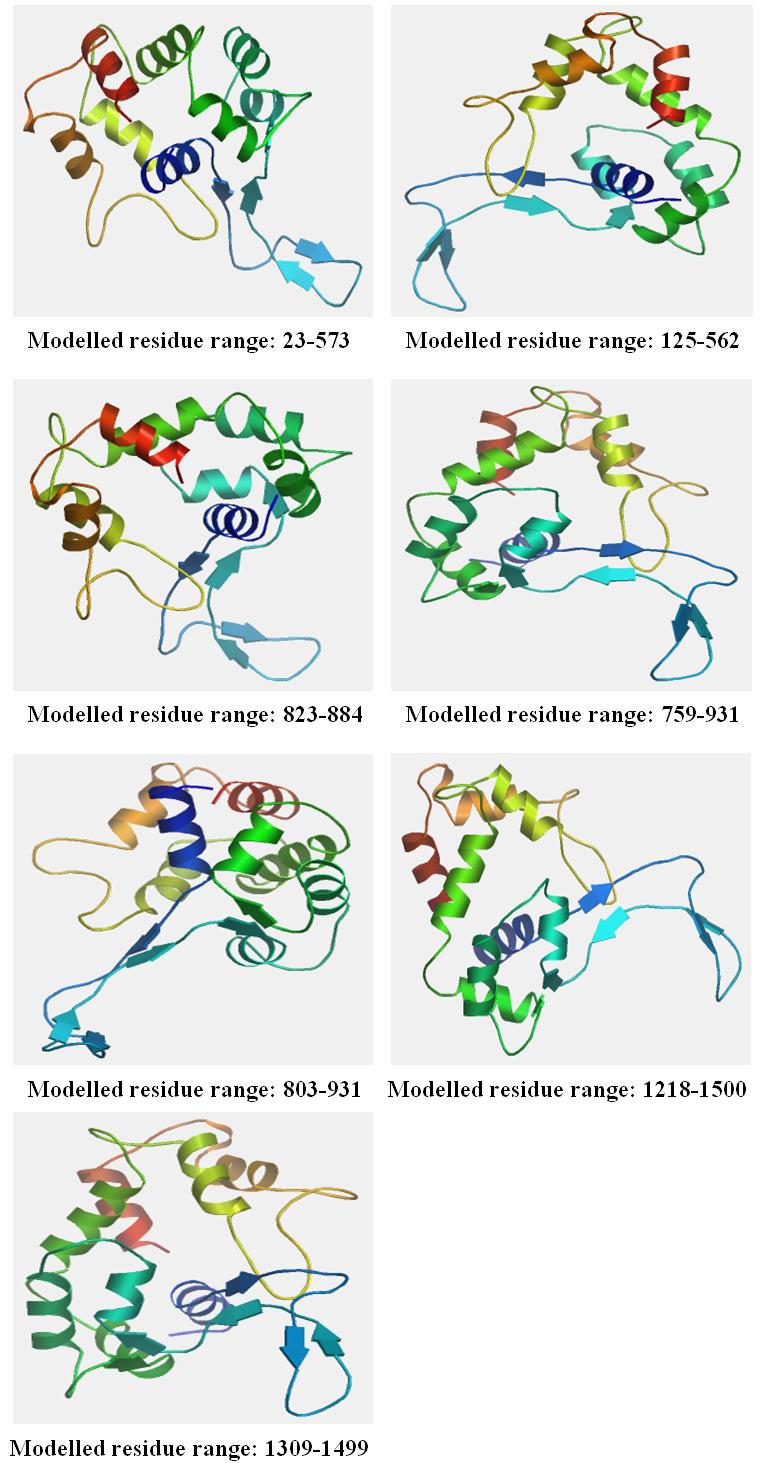

Supplement: Figure S4 — Tertiary structure of Bm Duox predicted by SWISS-MODEL. (TIF) [file pone.0070118.s004.tif]

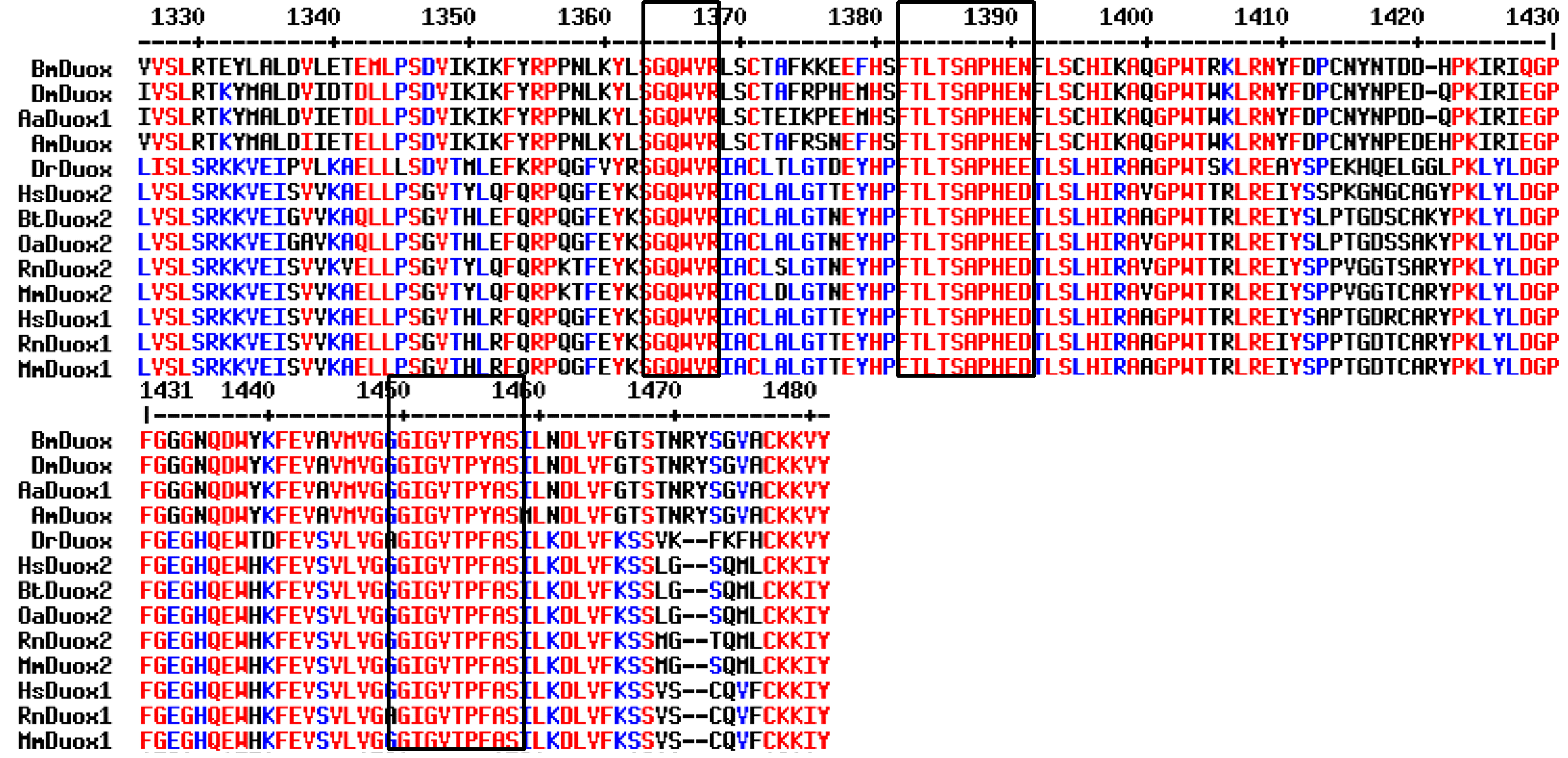

Supplement: Figure S6 — Comparison of NAD-binding domain amino acid sequence of Bm Duox with Duoxes of other species using the online multiple sequence alignment tool ( http://multalin.toulouse.inra.fr/multalin/multalin.html ). Red, high consensus; blue, low consensus; black, neutral consensus. Consensus levels: high, 90%; low, 50% must be less than the first value. (TIF) [file pone.0070118.s006.tif]

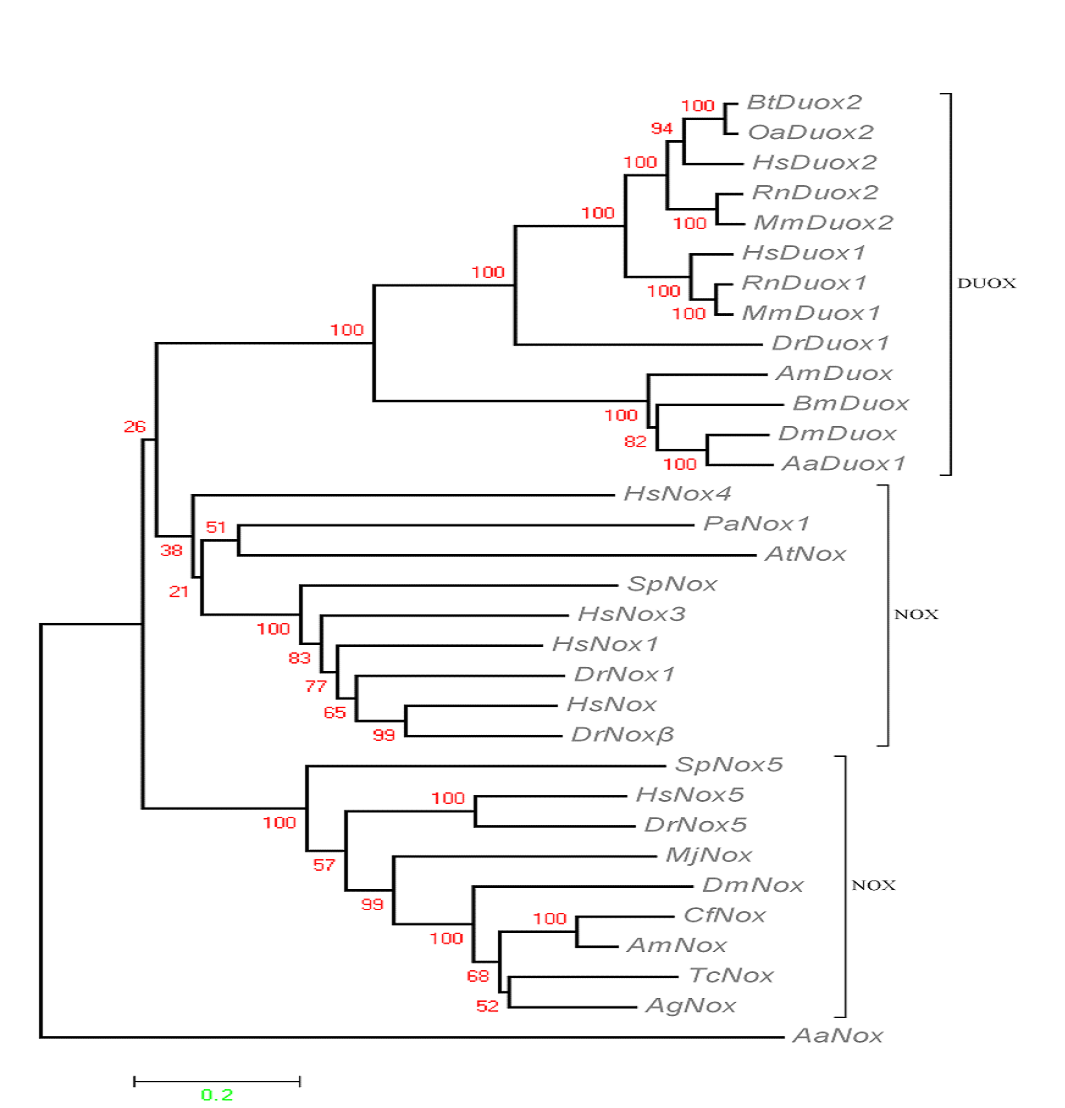

Supplement: Figure S7 — Phylogenetic relationship of the Duoxes and Noxes from the silkworm, other insects, vertebrates and plants. The deduced amino acid sequences were analyzed with ClustalW and MEGA 4 software. GenBank accession numbers of the other Duoxes/Noxes sequences are given in Table S2. (TIF) [file pone.0070118.s007.tif]

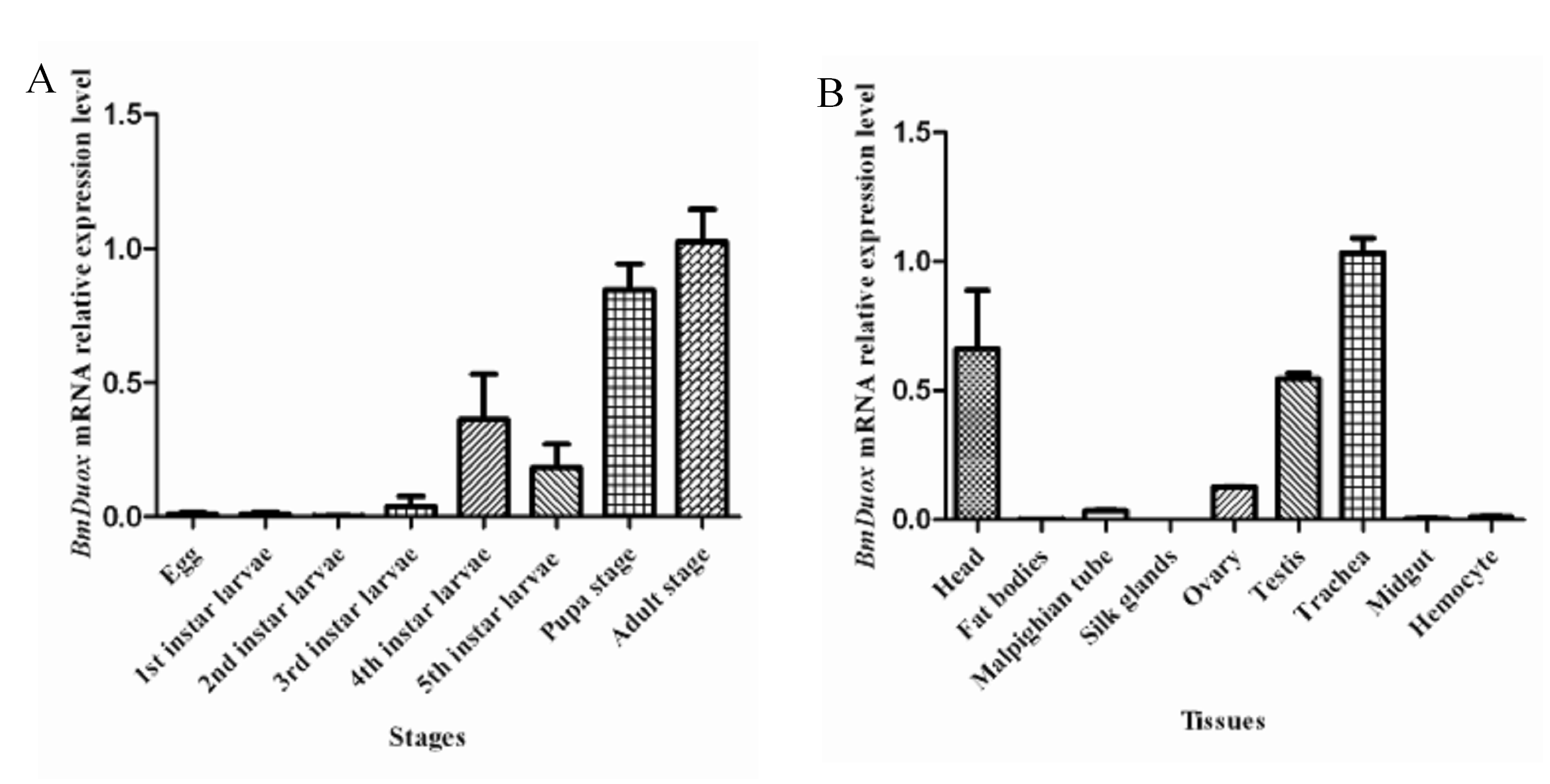

Supplement: Figure S8 — RT-PCR analysis of BmDuox expression in different developmental stages and different tissues. (A) Spatio-temporal distribution of BmDuox mRNA from egg to adult. (B) Tissue distribution of BmDuox mRNA in 3rd day of 5th instar larvae. The actin 3 gene was used as the internal control. (TIF) [file pone.0070118.s008.tif]

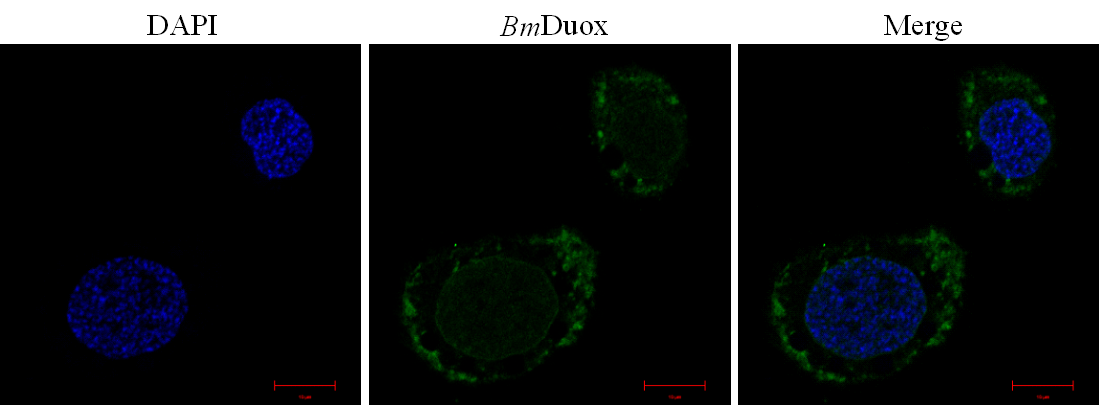

Supplement: Figure S9 — Subcellular localization of Bm Duox in BmN cells by immunofluorescence. BmN cells were fixed, probed with rabbit polyclonal BmDuox antibody to detect BmDuox, and visualized by FITC-labeled goat anti-rabbit IgG (green). Additionally, cells were stained with DAPI to visualize nuclear DNA (blue) directly. Cells were examined under a laser confocal scanning microscope (TCS SP5). (TIF) [file pone.0070118.s009.tif]

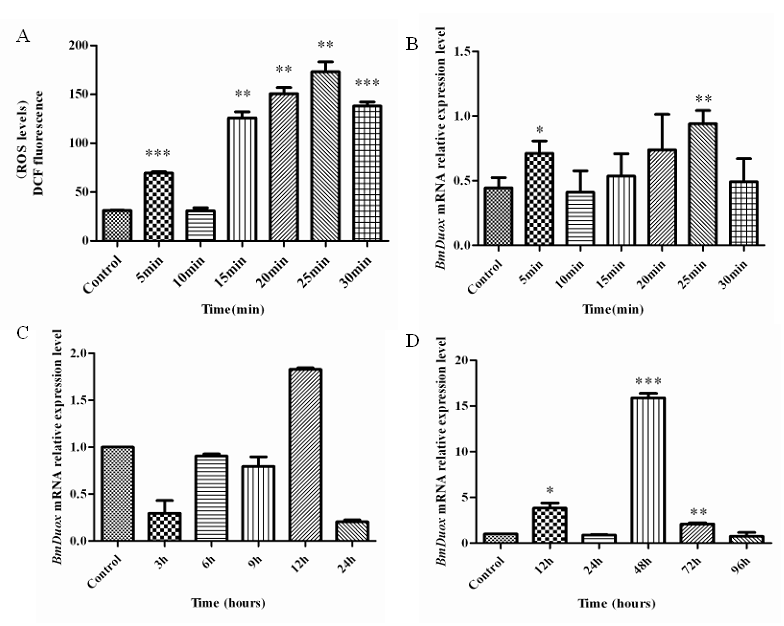

Supplement: Figure S10 — Induction expression of BmDuox and ROS generation in BmN cells and midgut. (A) ROS generation after SME treatment in BmN cells. (B) BmDuox expression pattern after SME treatment in BmN cells. (C) BmDuox expression pattern after DH5α treatment in the midgut. (D) BmDuox expression pattern after BmNPV treatment in the midgut. Results are expressed as the mean and standard deviation of three different experiments. Statistical differences were evaluated using Student’s t-test for unpaired samples. *P<0.05, **P<0.01, ***P<0.001. (TIF) [file pone.0070118.s010.tif]
