# Supplementary material for: Molecular Cloning and Functional Characterization of the Dual Oxidase (BmDuox) Gene from the Silkworm Bombyx mori
Source: PLoS One. 2013 Aug 2;8(8):e70118. doi: 10.1371/journal.pone.0070118 (PMC3732266; doi:10.1371/journal.pone.0070118)
Supplement: Table S1 — PCR primers used for BmDuox gene analysis. (DOC) [file pone.0070118.s011.doc]

**Table S1 PCR primers used for *Bombyx mori* *BmDuox* analysis.**

| Primers | Sequence (5’-3’) |
| --- | --- |
| 5’-RACE |  |
| 5’-DuoxR1 | GCATCTTCAGACCTCCTCCGCTTCTC |
| 5’-DuoxR2 | CGGATGCTGTTTGTGTACTCGGGCTGCG |
| Duox-Mid1  Duox-Mid2 | AGCATCCGGACTGGAGCGACGAACAACT  GCATCTTCAGACCTCCTCCGCTTCTC |
| 3’-RACE |  |
| 3’-DuoxF1 | GGCTACAGCTGAAACAAGGGAGCGTC |
| 3’-DuoxF2 | GACGCCGATGCCACCTCCCACCATCACC |
| BmDuox-expression-R | CTCGAGGCAAACTGGCTATGACAATCC |
| BmDuox-expression-F | GGATCCATGGCCGGTCCTGAGAGACCT |
|  |  |
| qRT-PCR analysis |  |
| Qduox-R | ACTGCCAACCGAACCCCAATC |
| Qduox-F | GCCGTTCTCTCTTCGTGCTAC |
| Qactin-F | AACACCCCGTCCTGCTCACTG |
| Qactin-R | GGGCGAGACGTGTGATTTCCT |
|  |  |
| Homologous recombinant |  |
| BmDuox-left-1 | TCTAGATCGTTGGTTAGTTAGGCTTTCAC |
| BmDuox-left-2 | AAGCTTGCAACCTTATCCACAAATCTACG |
| BmDuox-right-1 | GAATTCATGGACTGCCCTCACTAACGAAT |
| BmDuox-right-2 | GGATCCGAAGCGTTAGAAGTAGGAGTAGC |
| ie-1-*neo*-sv40polyA-1 | TCTAGAGATTTGCAGTTCGGGACATAA |
| ie-1-*neo*-sv40polyA-2 | TCTAGATAGCTAGAGGTCGACGGTATA |
| A3-*egfp*-sv40polyA-1 | CGCGTTACCATATATGGTGACA |
| A3-*egfp*-sv40polyA-2 | GCGATCCAGACATGATAAGAT |
| *egfp*-1 | GGATCCATGGTGAGCAAGGGCGAGGAG |
| *egfp*-2 | TCTAGATTACTTGTACAGCTCGTCCAT |
| ie-1-1 | AGATCTGATTTGCAGTTCGGGACATAA |
| ie-1-2 | AAGCTTAGTCGTTTGGTTGTTCACGAT |
