# Supplementary material for: Molecular Cloning and Functional Characterization of the Dual Oxidase (BmDuox) Gene from the Silkworm Bombyx mori
Source: PLoS One. 2013 Aug 2;8(8):e70118. doi: 10.1371/journal.pone.0070118 (PMC3732266; doi:10.1371/journal.pone.0070118)
Supplement: Table S2 — The symbols, GenBank accession numbers and nomenclature used in the phylogenetic analysis. (DOC) [file pone.0070118.s012.doc]

**Table S2 The symbols, GenBank accession numbers and nomenclatures used in the phylogenetic analysis.**

| Number | Symbol | Accession | Nomenclature |
| --- | --- | --- | --- |
| 1 | *Bm*Duox | JQ768349 | *Bombyx mori* |
| 2 | *Dm*Nox | NP_001097336.1 | *Drosophila melanogaster* |
| 3 | *Dm*Duox | NP_608715.2 | *Drosophila melanogaster* |
| 4 | *Cf*Nox | EFN67259 | *Camponotus floridanus* |
| 5 | *Aa*Nox | EAT37894.1 | *Aedes aegypti* |
| 6 | *Sp*Nox | NP_001073020.1 | *Strongylocentrotus purpuratus* |
| 7 | *Sp*Nox5 | XP_786060.2 | *Strongylocentrotus purpuratus5* |
| 8 | *Tc*Nox | XP_972375.2 | *Tribolium castaneum* |
| 9 | *Hs*Nox1 | CAI42337.1 | *Homo sapiens* |
| 10 | *Hs*Nox | AAH32720.1 | *Homo sapiens* |
| 11 | *Hs*Nox3 | NP_056533.1 | *Homo sapiens* |
| 12 | *Hs*Nox4 | AAF68973.1 | *Homo sapiens* |
| 13 | *Hs*Nox5 | ABC40743.1 | *Homo sapiens* |
| 14 | *Pa*Nox | AAK50853.1 | *Podospora anserina* |
| 15 | *At*Nox | O81211.2 | *Arabidopsis thaliana* |
| 16 | *Am*Nox | FAA00348.1 | *Apis mellifera* |
| 17 | *Ag*Nox | FAA00347.1 | *Anopheles gambiae* |
| 18 | *Dr*Duox | BAF33370.1 | *Danio rerio* |
| 19 | *Mj*Nox | AB594770.1 | *Marsupenaeus japonicus* |
| 20 | *Dr*Nox1 | NP_001095857 | *Danio rerio* |
| 21 | *Dr*Noxβ | NP_956708.1 | *Danio rerio* |
| 22 | *Dr*Nox5 | XP_001921894.1 | *Danio rerio* |
| 23 | *Hs*Duox2 | NP_054799.4 | *Homo sapiens* |
| 24 | *Hs*Duox1 | NP_059130.2 | *Homo sapiens* |
| 25 | *Rn*Duox2 | NP_077055.1 | *Rattus norvegicus* |
| 26 | *Rn*Duox1 | NP_714961.1 | *Rattus norvegicus* |
| 27 | *Mm*Duox2 | NP_808278.2 | *Mus musculus* |
| 28 | *Mm*Duox1 | NP_001092767.1 | *Mus musculus* |
| 29 | *Bt*Duox2 | XP_002690988.1 | *Bos taurus* |
| 30 | *Oa*Duox2 | NP_001177321.1 | *Ovis aries* |
| 31 | *Am*Duox | XP_624355.3 | *Apis mellifera* |
| 32 | *Aa*Duox1 | XP_001658452.1 | *Aedes aegypti* |
