# Supplementary material for: Molecular Cloning and Functional Characterization of the Dual Oxidase (BmDuox) Gene from the Silkworm Bombyx mori
Source: PLoS One. 2013 Aug 2;8(8):e70118. doi: 10.1371/journal.pone.0070118 (PMC3732266; doi:10.1371/journal.pone.0070118)
Supplement: Table S3 — Amino acid identity and similarity of the silkworm Bm Duox gene compared to other known Noxes/Duoxes sequences using each domain. Upper triangle, identity; lower triangle, similarity. A, Peroxidase domain; B, transmembrane region; C, calcium-binding region; D, ferric-reductase; E, FAD-binding domain; F, NAD-binding domain. (DOC) [file pone.0070118.s013.doc]

**Table S3 Amino acid identity and similarity of the silkworm BmDuox gene compared to other known Noxes/Duoxes sequences using each domain. Upper triangle: identity, lower triangle: similarity. A: Peroxidase domain, B: transmembrane region, C: calcium-binding region, D: Ferric-reduct, E: FAD-binding domai**n**, F: NAD-binding domain.**

| A Peroxidase domain | 1 | 2 | 3 | 4 | 5 | 6 | 7 | 8 | 9 | 10 | 11 | 12 | 13 |
| --- | --- | --- | --- | --- | --- | --- | --- | --- | --- | --- | --- | --- | --- |
| 1. *Bm*Duox |  | 42.7 | 67.3 | 41.7 | 41.9 | 40.0 | 41.7 | 40.9 | 41.1 | 39.8 | 39.6 | 62.3 | 68.6 |
| 2. *Dr*Duox | 64.0 |  | 42.1 | 54.0 | 53.4 | 54.7 | 54.2 | 54.4 | 53.0 | 54.4 | 54.2 | 41.4 | 42.5 |
| 3. *Dm*Duox | 83.0 | 65.0 |  | 41.1 | 42.0 | 40.4 | 41.1 | 40.4 | 40.9 | 40.8 | 40.8 | 63.5 | 78.6 |
| 4. *Hs*Duox2 | 63.3 | 71.2 | 63.5 |  | 83.6 | 83.7 | 78.6 | 84.4 | 77.7 | 86.5 | 86.7 | 41.2 | 43.0 |
| 5. *Hs*Duox1 | 60.9 | 72.4 | 61.4 | 90.7 |  | 77.3 | 86.8 | 76.7 | 87.0 | 77.3 | 77.3 | 40.7 | 41.6 |
| 6. *Rn*Duox2 | 62.0 | 71.0 | 63.5 | 92.2 | 87.7 |  | 82.2 | 94.1 | 80.3 | 81.6 | 81.6 | 39.6 | 41.3 |
| 7. *Rn*Duox1 | 61.4 | 71.6 | 61.2 | 87.7 | 92.4 | 90.7 |  | 81.3 | 94.7 | 77.3 | 77.3 | 40.4 | 41.8 |
| 8. *Mm*Duox2 | 62.7 | 71.6 | 63.7 | 93.2 | 87.9 | 98.3 | 90.4 |  | 82.4 | 82.3 | 82.5 | 40.7 | 41.3 |
| 9. *Mm*Duox1 | 61.2 | 71.6 | 61.2 | 87.5 | 92.8 | 90.5 | 97.7 | 90.7 |  | 77.3 | 77.3 | 39.2 | 40.8 |
| 10. *Bt*Duox2 | 61.6 | 71.2 | 63.5 | 91.6 | 86.6 | 90.5 | 86.6 | 90.7 | 87.5 |  | 97.0 | 39.7 | 41.3 |
| 11. *Oa*Duox2 | 61.0 | 70.5 | 63.1 | 91.6 | 85.6 | 90.3 | 85.6 | 90.5 | 86.4 | 98.1 |  | 39.4 | 41.5 |
| 12. *Am*Duox | 79.5 | 62.5 | 79.3 | 61.6 | 61.6 | 61.4 | 61.4 | 62.4 | 60.7 | 61.2 | 60.8 |  | 65.4 |
| 13. *Aa*Duox1 | 83.9 | 63.4 | 91.2 | 62.9 | 60.7 | 62.2 | 61.4 | 63.1 | 60.5 | 61.6 | 61.2 | 83.0 |  |

| B Transmembrane region | 1 | 2 | 3 | 4 | 5 | 6 | 7 | 8 | 9 | 10 | 11 | 12 | 13 |
| --- | --- | --- | --- | --- | --- | --- | --- | --- | --- | --- | --- | --- | --- |
| 1. *Bm*Duox |  | 24.1 | 57.3 | 32.4 | 30.1 | 30.8 | 30.1 | 30.8 | 30.1 | 33.8 | 33.8 | 47.9 | 71.4 |
| 2. *Dr*Duox | 50.7 |  | 25.2 | 49.3 | 61.4 | 53.5 | 57.1 | 54.2 | 57.1 | 52.8 | 53.5 | 27.6 | 30.6 |
| 3. *Dm*Duox | 67.7 | 49.1 |  | 31.7 | 35.0 | 32.1 | 34.4 | 31.7 | 34.4 | 37.2 | 36.6 | 53.6 | 75.5 |
| 4. *Hs*Duox2 | 54.3 | 75.4 | 51.6 |  | 72.5 | 81.4 | 71.1 | 85.5 | 71.1 | 92.8 | 92.8 | 30.0 | 36.6 |
| 5. *Hs*Duox1 | 53.6 | 80.4 | 55.9 | 87.7 |  | 71.4 | 93.5 | 70.4 | 94.2 | 76.1 | 76.1 | 30.4 | 38.1 |
| 6. *Rn*Duox2 | 53.6 | 77.5 | 51.6 | 95.7 | 89.1 |  | 70.0 | 91.4 | 70.0 | 80.7 | 80.0 | 31.8 | 34.3 |
| 7. *Rn*Duox1 | 52.9 | 78.3 | 55.9 | 86.2 | 96.4 | 86.2 |  | 68.3 | 99.3 | 73.9 | 73.9 | 32.7 | 38.1 |
| 8. *Mm*Duox2 | 54.3 | 76.1 | 52.8 | 98.6 | 87.7 | 95.7 | 85.5 |  | 68.3 | 84.1 | 84.8 | 31.2 | 34.5 |
| 9. *Mm*Duox1 | 52.9 | 78.3 | 55.9 | 85.5 | 97.1 | 86.2 | 99.3 | 84.8 |  | 73.9 | 73.9 | 32.7 | 38.1 |
| 10. *Bt*Duox2 | 53.6 | 77.5 | 52.2 | 97.8 | 89.1 | 94.9 | 87.0 | 97.8 | 87.0 |  | 99.3 | 31.2 | 38.7 |
| 11. *Oa*Duox2 | 53.6 | 77.5 | 52.2 | 97.8 | 89.1 | 94.9 | 87.0 | 97.8 | 87.0 | 100 |  | 31.2 | 38.7 |
| 12. *Am*Duox | 64.9 | 53.4 | 70.2 | 54.7 | 56.1 | 53.4 | 56.1 | 53.4 | 56.1 | 54.1 | 54.1 |  | 54.1 |
| 13. *Aa*Duox1 | 81.9 | 55.8 | 79.5 | 60.1 | 61.6 | 59.4 | 60.9 | 59.4 | 60.9 | 60.9 | 60.9 | 72.3 |  |

| C Calcium bindging motif | 1 | 2 | 3 | 4 | 5 | 6 | 7 | 8 | 9 | 10 | 11 | 12 | 13 |
| --- | --- | --- | --- | --- | --- | --- | --- | --- | --- | --- | --- | --- | --- |
| 1. *Bm*Duox |  | 41.4 | 89.7 | 46 | 48.3 | 47.1 | 48.3 | 47.1 | 48.3 | 34.5 | 33.3 | 88.5 | 89.7 |
| 2. *Dr*Duox | 71.3 |  | 36.8 | 59.8 | 59.8 | 60.9 | 59.8 | 59.8 | 59.8 | 47.1 | 46 | 41.4 | 36.8 |
| 3. *Dm*Duox | 95.4 | 70.1 |  | 43.7 | 43.7 | 44.8 | 43.7 | 44.8 | 43.7 | 33.3 | 32.2 | 85.1 | 100 |
| 4. *Hs*Duox2 | 75.9 | 89.7 | 73.6 |  | 90.7 | 96.5 | 90.7 | 97.7 | 90.7 | 65.1 | 64 | 42.5 | 43.7 |
| 5. *Hs*Duox1 | 77 | 87.4 | 74.7 | 95.3 |  | 89.5 | 100 | 90.7 | 100 | 58.1 | 57 | 44.8 | 43.7 |
| 6. *Rn*Duox2 | 75.9 | 90.8 | 73.6 | 98.8 | 96.5 |  | 89.5 | 98.8 | 89.5 | 64 | 62.8 | 44.8 | 44.8 |
| 7. *Rn*Duox1 | 77 | 87.4 | 74.7 | 95.3 | 100 | 96.5 |  | 90.7 | 100 | 58.1 | 57 | 44.8 | 43.7 |
| 8. *Mm*Duox2 | 75.9 | 90.8 | 73.6 | 98.8 | 96.5 | 100 | 96.5 |  | 90.7 | 65.1 | 64 | 43.7 | 44.8 |
| 9. *Mm*Duox1 | 77 | 87.4 | 74.7 | 95.3 | 100 | 96.5 | 100 | 96.5 |  | 58.1 | 57 | 44.8 | 43.7 |
| 10. *Bt*Duox2 | 50.6 | 63.2 | 48.3 | 66.3 | 64 | 67.4 | 64 | 67.4 | 64 |  | 98.3 | 32.2 | 33.3 |
| 11. *Oa*Duox2 | 49.4 | 62.1 | 47.1 | 65.1 | 62.8 | 66.3 | 62.8 | 66.3 | 62.8 | 98.3 |  | 31 | 32.2 |
| 12. *Am*Duox | 93.1 | 71.3 | 93.1 | 77 | 78.2 | 77 | 78.2 | 77 | 78.2 | 50.6 | 49.4 |  | 85.1 |
| 13.*Aa*Duox1 | 95.4 | 70.1 | 100 | 73.6 | 74.4 | 73.6 | 74.4 | 73.6 | 74.7 | 48.3 | 47.1 | 93.1 |  |

| D Ferric-reduct | 1 | 2 | 3 | 4 | 5 | 6 | 7 | 8 | 9 | 10 | 11 | 12 | 13 |
| --- | --- | --- | --- | --- | --- | --- | --- | --- | --- | --- | --- | --- | --- |
| 1. *Bm*Duox |  | 39.5 | 77.9 | 44.1 | 43 | 40.8 | 43.7 | 42.1 | 43.7 | 43.4 | 43.4 | 76.5 | 83.2 |
| 2. *Dr*Duox | 66.0 |  | 41.3 | 66.7 | 69.3 | 68.7 | 68.0 | 68.0 | 68.0 | 67.3 | 67.3 | 40.7 | 39.3 |
| 3. *Dm*Duox | 88.6 | 64.7 |  | 42.0 | 44.0 | 41.1 | 43.3 | 41.7 | 43.3 | 42.0 | 42.0 | 78.4 | 88.5 |
| 4. *Hs*Duox2 | 69.3 | 88.0 | 62.7 |  | 84.0 | 89.3 | 82.7 | 90.7 | 82.7 | 98.0 | 98.0 | 42.0 | 43.0 |
| 5. *Hs*Duox1 | 68.7 | 91.3 | 66.7 | 94 |  | 80.0 | 93.3 | 80.7 | 94.7 | 85.3 | 85.3 | 43.3 | 44.0 |
| 6. *Rn*Duox2 | 68.7 | 88.0 | 64.7 | 97.3 | 93.3 |  | 80.0 | 96.0 | 80.0 | 88.7 | 88.7 | 42.7 | 41.1 |
| 7. *Rn*Duox1 | 69.3 | 90.0 | 66.0 | 95.3 | 98.0 | 92.0 |  | 82.0 | 98.7 | 83.3 | 83.3 | 43.3 | 42.0 |
| 8. *Mm*Duox2 | 68.7 | 88.7 | 65.3 | 98.0 | 93.3 | 98.0 | 94.0 |  | 82.0 | 90.0 | 90.0 | 42.0 | 41.1 |
| 9. *Mm*Duox1 | 69.3 | 90.0 | 66.0 | 94.0 | 98.7 | 91.3 | 99.3 | 92.7 |  | 83.3 | 83.3 | 43.3 | 42.0 |
| 10. *Bt*Duox2 | 69.3 | 89.3 | 64.0 | 98.7 | 95.3 | 96.0 | 96.0 | 98.0 | 95.3 |  | 100 | 41.3 | 42.4 |
| 11. *Oa*Duox2 | 69.3 | 89.3 | 64.0 | 98.7 | 95.3 | 96.0 | 96.0 | 98.0 | 95.3 | 100 |  | 41.3 | 42.4 |
| 12. *Am*Duox | 88.6 | 68.0 | 88.5 | 69.3 | 70.7 | 68.7 | 68.7 | 67.3 | 68.7 | 69.3 | 69.3 |  | 78.4 |
| 13. *Aa*Duox1 | 91.3 | 64.7 | 93.2 | 67.3 | 68.0 | 67.3 | 66.7 | 66.7 | 66.7 | 67.3 | 67.3 | 91.2 |  |

| E FAD-binding domain | 1 | 2 | 3 | 4 | 5 | 6 | 7 | 8 | 9 | 10 | 11 | 12 | 13 |
| --- | --- | --- | --- | --- | --- | --- | --- | --- | --- | --- | --- | --- | --- |
| 1. *Bm*Duox |  | 47.1 | 86.3 | 47.6 | 46.6 | 46.6 | 47.6 | 46.6 | 47.6 | 45.6 | 44.7 | 88.3 | 88.2 |
| 2. *Dr*Duox | 64.4 |  | 46.2 | 76.0 | 75.0 | 72.1 | 76.0 | 73.1 | 76.0 | 76.0 | 74.0 | 46.2 | 46.2 |
| 3. *Dm*Duox | 95.1 | 62.5 |  | 47.6 | 46.6 | 46.6 | 46.6 | 46.6 | 46.6 | 46.6 | 45.6 | 92.2 | 94.1 |
| 4. *Hs*Duox2 | 67.0 | 82.7 | 65.0 |  | 92.2 | 89.3 | 92.2 | 91.3 | 92.2 | 88.3 | 86.4 | 46.6 | 46.6 |
| 5. *Hs*Duox1 | 67.0 | 80.8 | 65.0 | 96.1 |  | 87.4 | 98.1 | 89.3 | 98.1 | 92.2 | 88.3 | 45.6 | 45.6 |
| 6. *Rn*Duox2 | 68.0 | 81.7 | 66.0 | 91.3 | 91.3 |  | 89.3 | 97.1 | 89.3 | 84.5 | 84.5 | 46.6 | 46.6 |
| 7. *Rn*Duox1 | 68.9 | 81.7 | 67.0 | 95.1 | 98.1 | 93.2 |  | 91.3 | 100 | 92.2 | 88.3 | 46.6 | 45.6 |
| 8. *Mm*Duox2 | 66.0 | 81.7 | 65.0 | 92.2 | 92.2 | 97.1 | 94.2 |  | 91.3 | 86.4 | 84.5 | 46.6 | 46.6 |
| 9. *Mm*Duox1 | 68.9 | 81.7 | 67.0 | 95.1 | 98.1 | 93.2 | 100 | 94.2 |  | 92.2 | 88.3 | 46.6 | 45.6 |
| 10. *Bt*Duox2 | 67.0 | 82.7 | 65.0 | 93.2 | 95.1 | 92.2 | 96.1 | 93.2 | 96.1 |  | 96.1 | 45.6 | 45.6 |
| 11. *Oa*Duox2 | 67.0 | 80.8 | 64.1 | 91.3 | 91.3 | 92.2 | 92.2 | 91.3 | 92.2 | 96.1 |  | 45.6 | 44.7 |
| 12. *Am*Duox | 95.1 | 66.3 | 97.1 | 68.9 | 68.9 | 69.9 | 69.9 | 68.9 | 69.9 | 69.9 | 68.9 |  | 89.3 |
| 13. *Aa*Duox1 | 94.1 | 64.4 | 97.1 | 65.0 | 65.0 | 65.0 | 66.0 | 66.0 | 66.0 | 64.1 | 63.1 | 94.2 |  |

| F NAD-binding domain | 1 | 2 | 3 | 4 | 5 | 6 | 7 | 8 | 9 | 10 | 11 | 12 | 13 |
| --- | --- | --- | --- | --- | --- | --- | --- | --- | --- | --- | --- | --- | --- |
| 1. *Bm*Duox |  | 54.8 | 96.2 | 52.9 | 54.1 | 54.1 | 54.1 | 54.1 | 54.1 | 54.1 | 54.1 | 93 | 96.8 |
| 2. *Dr*Duox | 76.4 |  | 55.4 | 82.6 | 84.5 | 83.9 | 85.2 | 83.2 | 84.5 | 83.9 | 83.9 | 54.1 | 55.4 |
| 3. *Dm*Duox | 99.4 | 76.4 |  | 53.5 | 54.8 | 54.8 | 54.8 | 54.8 | 54.8 | 54.8 | 54.8 | 95.5 | 99.4 |
| 4. *Hs*Duox2 | 75.8 | 92.3 | 76.4 |  | 95.5 | 92.3 | 92.9 | 96.1 | 94.8 | 96.1 | 96.1 | 53.5 | 53.5 |
| 5. *Hs*Duox1 | 75.8 | 92.3 | 76.4 | 98.7 |  | 91.6 | 97.4 | 94.8 | 99.4 | 94.2 | 94.2 | 54.8 | 54.8 |
| 6. *Rn*Duox2 | 75.2 | 91 | 75.8 | 97.4 | 96.1 |  | 92.9 | 94.2 | 91.6 | 92.9 | 92.9 | 53.5 | 54.8 |
| 7. *Rn*Duox1 | 75.2 | 92.9 | 75.8 | 97.4 | 98.7 | 96.1 |  | 92.9 | 97.4 | 92.3 | 92.3 | 53.5 | 54.8 |
| 8. *Mm*Duox2 | 75.2 | 91.6 | 75.8 | 98.1 | 96.8 | 97.4 | 96.1 |  | 94.8 | 96.8 | 96.8 | 54.8 | 54.8 |
| 9. *Mm*Duox1 | 75.8 | 92.3 | 76.4 | 98.1 | 99.4 | 96.1 | 98.7 | 96.8 |  | 94.2 | 94.2 | 54.8 | 54.8 |
| 10. *Bt*Duox2 | 75.8 | 92.9 | 76.4 | 98.7 | 97.4 | 98.1 | 96.8 | 98.7 | 97.4 |  | 98.7 | 54.8 | 54.8 |
| 11. *Oa*Duox2 | 75.8 | 91.6 | 76.4 | 98.1 | 96.8 | 96.8 | 96.1 | 98.1 | 96.8 | 99.4 |  | 54.8 | 54.8 |
| 12. *Am*Duox | 98.7 | 77.1 | 99.4 | 76.4 | 76.4 | 75.8 | 75.8 | 75.8 | 76.4 | 76.4 | 76.4 |  | 94.9 |
| 13. *Aa*Duox1 | 99.4 | 76.4 | 100 | 76.4 | 76.4 | 75.8 | 75.8 | 75.8 | 76.4 | 76.4 | 76.4 | 99.4 |  |
